# SozRank: A new approach for localizing the epileptic seizure onset zone

**DOI:** 10.1371/journal.pcbi.1005953

**Published:** 2018-01-30

**Authors:** Yonathan Murin, Jeremy Kim, Josef Parvizi, Andrea Goldsmith

**Affiliations:** 1 Department of Electrical Engineering, Stanford University, Stanford, CA, United States of America; 2 Department of Neurology & Neurological Sciences, Stanford University, Stanford, CA, United States of America; University of Maryland at College Park, UNITED STATES

## Abstract

Epilepsy is one of the most common neurological disorders affecting about 1% of the world population. For patients with focal seizures that cannot be treated with antiepileptic drugs, the common treatment is a surgical procedure for removal of the seizure onset zone (SOZ). In this work we introduce an algorithm for automatic localization of the seizure onset zone (SOZ) in epileptic patients based on electrocorticography (ECoG) recordings. The proposed algorithm builds upon the hypothesis that the abnormal excessive (or synchronous) neuronal activity in the brain leading to seizures *starts in the SOZ* and then *spreads* to other areas in the brain. Thus, when this abnormal activity starts, signals recorded at electrodes close to the SOZ should have a relatively large *causal influence* on the rest of the recorded signals. The SOZ localization is executed in two steps. First, the algorithm represents the set of electrodes using a directed graph in which nodes correspond to recording electrodes and the edges’ weights quantify the *pair-wise* causal influence between the recorded signals. Then, the algorithm infers the SOZ from the estimated graph using a variant of the *PageRank* algorithm followed by a novel post-processing phase. Inference results for 19 patients show a close match between the SOZ inferred by the proposed approach and the SOZ estimated by expert neurologists (success rate of 17 out of 19).

## Author summary

Epilepsy is a common neurological disorder characterized by abnormal electrical disturbances in the brain that result in transient occurrence of signs and/or symptoms, also known as seizures. In focal epilepsy, this electrical activity originates from a limited area in the brain, commonly referred to as the *seizure onset zone* (SOZ). For patients with focal epilepsy that cannot be treated with medications, the common treatment is a resective surgery to remove the SOZ. This work presents an algorithm for SOZ localization based on electrocorticography recordings. Such an automatic solution has the potential to increase the localization accuracy, to provide a validation of the neurologist’s SOZ region, and to ultimately reduce or eliminate the analysis time of the neurologist. Inference results for 19 patients show a close match between the SOZ inferred by the proposed algorithm and the SOZ estimated by expert neurologists.

## Introduction

Epilepsy is one of the most common neurological disorders affecting about 70 million people worldwide. It is characterized by recurrent episodes of abnormal neural activity in the central nervous system [1]. This activity leads to transient occurrence of signs and/or symptoms, also known as epileptic seizures. The clinical symptoms of epileptic seizures range from auras, to spasmodic muscular contractions, up to loss of consciousness [2, 3]. Epileptic seizures can be roughly divided into two groups, based on the location in the brain from which the abnormal neural activity originates and how it propagates. In partial, or focal, seizures the abnormal neural activity originates from a limited area in the brain, commonly referred to as the *seizure onset zone* (SOZ). On the other hand, primary generalized seizures begin with a widespread electrical discharge that involves most of the brain. In this work we consider focal epilepsy and present an algorithm for SOZ localization, that is, *determining the area in the brain where the abnormal neural activity leading to a focal seizure originates* (a guiding hypothesis throughout our work is that in focal seizures there is a singular focal point, from which this activity originates).

The common and simplest approach to treat epilepsy is using antiepileptic drugs. Yet, in about 25–33% of the patients this approach is not effective [4], and a patient is diagnosed with *refractory epilepsy*. A possible treatment approach for refractory epilepsy is a resective surgery procedure to remove the areas in the brain that are *necessary and sufficient* to generate the abnormal neural activity that leads to epileptic seizures. Currently, it is not known how these areas, also referred to as the *epileptogenic zone* (EZ), can be mapped. Therefore, in clinical practice, the SOZ is used as an approximation for the EZ [5], and in the resective surgery the estimated SOZ is removed (assuming this region is not responsible for indispensable brain functions). Recent longitudinal trials indicate that long-term seizure freedom can be achieved in up to two thirds of the patients who undergo surgery [6]. The main tool for SOZ identification (localization), in cases where the SOZ is not evident in a non-invasive electrocorticography (EEG) or in an MRI, is invasive EEG (also known as electrocorticography (ECoG)). In ECoG grids or strips of electrodes are placed on the cortex [2], allowing a direct measurement and recording of the brain’s electrical activity (local field potentials). These recordings, together with video monitoring, are used by expert neurologists to approximate the electrodes associated with the area within which the SOZ lies. In this paper we describe an algorithm that localizes the SOZ based on the ECoG recordings. Such an *automated* solution will provide a valuable tool for neurologists to assist in SOZ localization and perhaps increase localization accuracy over current methods.

The algorithm proposed in this paper builds upon a fundamental property of focal seizures reported in [7]: *the abnormal neural activity associated with focal seizures starts in the SOZ and spreads to other areas in the brain*. Therefore, at the beginning of such activity, signals recorded at electrodes located in vicinity of the SOZ should have a relatively large causal influence on the rest of the recorded signals. This calls for an algorithm that estimates and incorporates the causal influence between the different recorded signals into its SOZ localization. Since the electrodes in an ECoG grid are relatively close together [8], the signals recorded in the different electrodes are statistically dependent. In such a case, to fully quantify the statistical causal influence between two electrodes, one must evaluate this influence *when conditioning on the rest of the electrodes* [9, 10]. Unfortunately, even for moderate-size grids with 16 electrodes, this task is *too computationally demanding* and requires a *huge amount of data* per each seizure. Therefore, in this paper we take a different path and *approximate* the underlying causal influence structure. Instead of (statistically) conditioning on the rest of the recordings, the proposed algorithm applies a practical approximation by considering the electrodes as nodes in a *directed graph*, where the edges’ weights are estimations of the *pair-wise causal influences*. In this work we focus on the following question: *how should the SOZ be inferred from the estimated graph?* The procedure for estimating the graph is discussed in S1 Text.

The method of representing causal influences among a set of random variables using directed graphs is not new. In [11] this approach was used while adding the constraint that the graph should be acyclic. In this case it is assumed that the joint density follows a causal Markov condition [11]. However, with ECoG recordings the Markovian structure of the underlying density is not known, and it must be estimated from the recordings. A possible approach for estimating this structure is via minimizing the KL divergence [12, Sec. 8.5] between the true density and an approximated density induced by a spanning tree. As shown in [13], the best tree (in terms of minimizing the KL divergence) can be found using the maximum-weight spanning tree algorithm (Edmonds’ algorithm) [14]. Moreover, the underlying hypothesis for localization based on this approach is that the root of the spanning tree should correspond to the origin of the causal activity. This localization approach was taken in [15], which our work improves upon in multiple dimensions. In particular, [15] assumes that the underlying density follows a specific structure in order to apply Edmonds’ algorithm. However, it is not clear if this structural assumption accurately describes the observed signals. In addition, the algorithm of [15] localizes the SOZ using only the outgoing weights, whereas other works [16, 17] use both incoming and outgoing node weights. Hence, our work contrasts with [15] by using the PageRank algorithm to account for the structure of the estimated graph rather than assuming a specific structure, and basing our localization on both the incoming and outgoing weights to each node.

Another approach for inferring the SOZ from the estimated graph was proposed in [16, 17]: based on the findings of [7], the nodes in the SOZ should have properties of “sources of causal influence” with large outgoing flow and small incoming flow (the total incoming flow subtracted from the total outgoing flow is referred to as the net-flow). The two main drawbacks of this approach is that it ignores the *structure* of the estimated graph, and ranks the nodes (electrodes) based only on their one-step neighbors. Our study shows that for some of the patients this approach works well, while for others the results can be improved by a more sophisticated inference approach.

To account for the *structure* of the graph (and for multi-step neighbors), we propose to use a variant of Google’s famous PageRank algorithm [18, 19]. The PageRank algorithm, initially designed for *ranking web pages*, is based on the following thesis [20]: *A web page is important if it is pointed to by other important pages*. Motivated by this thesis, the PageRank algorithm views the web as a directed graph with pages as nodes and hyperlinks as edges, and ranks the web pages based on the steady-state probability of a *random surfer* visiting each page. Using terminology taken from another web ranking algorithm, the hyperlink-induced topic search (HITS) algorithm [21], the PageRank algorithm can also be viewed as assigning *authority* scores to the nodes. A high *authority* score is given to a page that is *linked by many other pages with high authority scores*. Thus, we use PageRank to calculate an in-flow (authority) score for each node in the graph. To calculate an out-flow score we use the Reverse PageRank algorithm [22]. As PageRank ranks based on the dominant right eigenvector of the directed graph, it accounts for its structure. We emphasize that in our algorithm the PageRank *does not model the propagation of the abnormal neural activity*. Instead, it is used to *evaluate the importance* of a node in terms of its causal influence on the rest of the network. It should further be noted that the PageRank algorithm was already used in the context of neuroscience problems. For example, [23] studied the network architecture of functional connectivity within the human brain connectum, and used four centrality measures, of which PageRank was one, to provide insights on this connectivity.

Numerous works have studied the problem of localizing the SOZ using ECoG recordings. We refer the reader to [2, 16] and references therein for background on this topic. Many of the algorithms proposed in previous studies are based on some form of a (causal) connectivity graph and use the following three main steps: *i*) Pre-processing the ECoG recorded signals; *ii*) Estimating the connectivity graph from the processed ECoG signals; and *iii*) Inferring the SOZ from the estimated connectivity graph. While the algorithm proposed in the current paper follows a similar approach, it uses an improved method to estimate the connectivity graph and applies a novel method to infer the SOZ from it. Specifically, the proposed algorithm analyzes two types of 10 seconds blocks: at the beginning of a seizure (an ictal block) as well as blocks randomly sampled when the patient is resting (rest blocks). By using information from both types of blocks, the proposed algorithm accounts for the structure of the estimated network when no seizure is evolving. The value of 10 seconds was chosen to provide a good tradeoff between the number of samples in a block and the stationarity of the observed signals over a block. When the block is much longer than 10 seconds the data may not be stationary, while when the block is much shorter than 10 seconds the number of samples available for estimating the pair-wise causal influences is too small (the estimation may not be accurate enough). In contrast to the proposed algorithm, previous studies used significantly longer blocks [16, 17]. To quantify the pair-wise causal influences between the recordings, the proposed algorithm uses a combination of a parametric causality measure, Granger causality [24], and a non-parametric measure, directed information [25]. Our results show that this combined procedure improves upon using each of the above approaches (parametric or non-parametric) separately. Finally, the proposed algorithm uses a novel approach to infer the SOZ from the estimated graph. In particular, by using a variation of the PageRank algorithm, a score is assigned to each node. The algorithm then selects the SOZ nodes as the nodes that have high scores compared to other nodes, as well as compared to scores calculated *based on the rest blocks*. We emphasize that previous studies [16, 17] did not account for the rest blocks as part of the localization procedure. Our analysis, on the other hand, suggests that rest block should be taken into account when localizing the SOZ in order to avoid biased results.

## Results

Despite the advances in automated SOZ localization described above, the gold standard is still considered to be the localization performed by expert neurologists. Hence, to evaluate the performance of our proposed localization algorithm, we compare its inferences with the inference made by these experts. We show that for 17 out of 19 patients with refractory epilepsy, all listed in the International Epilepsy Electrophysiology (iEEG) portal [26], the inferences of our algorithm closely align with the inference performed by the neurologists (see the exact definitions below for the success rate and average false positive detection rate of the proposed algorithm). Moreover, even for the two patients for which we do not have a complete match, the inference made by the proposed algorithm is strongly correlated with the SOZ estimated by expert neurologists or with the seizure evolution (see the discussion at the end of this section). Hence, these inferences can be viewed as a *partial success*. In the Discussion section we test our algorithm against other algorithms to demonstrate its superior performance.

### Description of the tested data

The proposed algorithm was tested on 19 data-sets, taken from patients undergoing surgical treatment for medically refractory epilepsy. These data-sets are listed on the online iEEG portal [26] (http://www.ieeg.org). The patient-specific information is detailed in Table 1. The ECoG signals were sampled at rates between 500 Hz and 5 KHz: data-set I001_P034_D01 was sampled at 5 KHz (Mayo Clinic, Rochester, MN). Data-sets Study_004-2—Study_037 were sampled at 500 Hz (Mayo Clinic, Rochester, MN), and data-sets HUP64_phaseII—HUP87_phaseII (Hospital of the University of Pennsylvania, Philadelphia, PA) were sampled at 512 Hz.

**Table 1:** Patient information. ALT—Anterior left temporal, BL—Bilateral left, RF—Right frontal, RT—Right temporal, LO—Left occipital, LF—Left frontal, LP—Left perirolandic, MTS—Mesial temporal sclerosis, CP—complex-partial, CPG—complex partial with secondary generalization, GA—Generalized atonic, SP—Simple partial, NF—No follow-up, NR—No resection.

Patient (iEEG Portal)	Sex	Age (Onset/Surgery)	Seizure Onset	Seizure Type	#Seizures	Grid Size	Grid Name	Outcome Class
I001_P034_D01	F	Unknown	RF	CPG	16	6 × 6	GRID	NF
Study_004-2	F	14/27	RT ccipital	CPG	3	6 × 6	RG	IV
Study_006	M	22/25	RF	CP	5	6 × 8	LG	NR
Study_010	F	00/13	LF	CP	3	6 × 8	GRID	NF
Study_016	F	05/36	RT orbitofrontal	CPG	4	4 × 6	RTG	IV
Study_017	F	Unknown	Unknown	CPG	5	1 × 8	RTD	IV
Study_020	M	05/10	RF	CPG	8	4 × 6	RAG	IV
Study_021	M	Unknown	RF	CPG	13	6 × 8	RFG	I
Study_022	F	Unknown	Unknown	CPG	7	4 × 6	TIG	V
Study_023	M	01/16	LO	CP	4	8 × 8	LTG	I
Study_027	M	Unknown	Unknown	CPG	6	3 × 8	LG	NF
Study_033	M	00/03	LF	GA	17	8 × 8	LG	V
Study_037	F	Unknown	Unknown	CP	8	8 × 8	RPG	NR
HUP64_phaseII	F	03/20	LF	CPG	1	8 × 8	LG	I
HUP65_phaseII	M	02/36	RT	CPG	3	8 × 8	RG	I
HUP68_phaseII	F	15/26	RT	CP, CPG	5	8 × 8	RG	I
HUP70_phaseII	M	10/32	LP	SP	8	8 × 8	RG	NR
HUP78_phaseII	M	00/54	ALT	CP	5	8 × 8	LG	III
HUP87_phaseII	M	21/24	Frontal	CP	2	8 × 8	LG	I

Each of the data-sets contains ECoG recordings, as well as annotations indicating which time intervals in the recordings correspond to seizures. The video recordings are used to generate these annotations. The data-sets also include reports describing the spatial locations, on the cortex, of the electrodes, and comments by expert neurologists as to where the seizures originate from. We refer to an electrode that is highlighted in these comments as an *electrode of interest* (EOI). Some of these data-sets contain recordings from several strips and grids. In these cases, the proposed algorithm analyzed the largest grid of electrodes (in all considered cases the largest grid was located over the suspected SOZ). The name of the analyzed grid and its size are specified in Table 1. Table 1 also specifies the surgical outcome (class) for each patient. Note that three patients were not resected, and there is no follow-up for three other patients.

### Summary of the localization results

**Fig 1:**
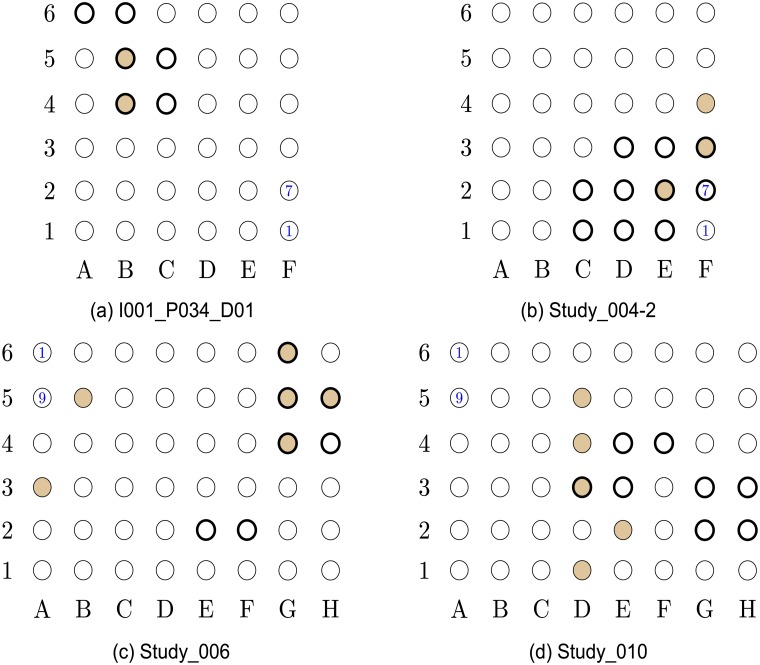
Localization results for data-sets I001_P034_D01 to Study_010. The EOI nodes are marked by a *bold annulus*, whereas the nodes detected by our proposed algorithm are marked by solid brown circles. (a) (*Top left*) Localization results for data-set I001_P034_D01. This is a successful localization, with *V*_p_ = 0. Note that in in this data-set, even though node 6A–6B are mentioned in the report, their recordings are missing from the data-set. (b) (*Top right*) Localization results for data-set Study_004-2. This is a successful localization, with *V*_p_ = 0. (c) (*Bottom left*) Localization results for data-set Study_006. This is a successful localization, with *V*_p_ = 0.069. (d) (*Bottom right*) Localization results for data-set Study_010. This is a successful localization, with *V*_p_ = 0.071.

We summarize the localization results using the following two metrics:

1. **Success rate**: We say that an inference is *successful* if more than 50% of the inferred electrodes (nodes) overlap with the EOI (for the specific patient), or with the nodes *strictly adjacent* to the EOI. For instance, node 5A in Fig 1-(a) is adjacent to the EOI, while nodes 3A or 2B are not. Using this definition the success rate of the proposed algorithm is 17 out of 19.
2. **Average false positive detection rate**: We say that an inferred node is a *false-positive detection* if it is not part of the EOI or adjacent nodes. For example, node 5B in Fig 1-(c) is a false-positive detection. To calculate the *false-positive detection rate* for a patient *p*, denoted by *V*_p_, we count the number of detected false-positive inferences, and divide it by the number of nodes that *are not part of the EOI or its adjacent nodes*. Extending the example, in Fig 1-(c) nodes {1E–1F, 2D–2G, 3E–3H, 4F–4H, 5F–5H, 6T–6H} constitute the set of EOI and adjacent nodes. Therefore, as our algorithm inferred 2 nodes out of this group (see 3A and 5B), the *false-positive detection rate* is 0.069. The *average false-positive detection rate* is the false-positive detection rate averaged over all patients. The proposed algorithm achieves *average false-positive detection rate* of 0.03.

Next, we provide a detailed description of our localization results.

### Detailed localization results

**Fig 2:**
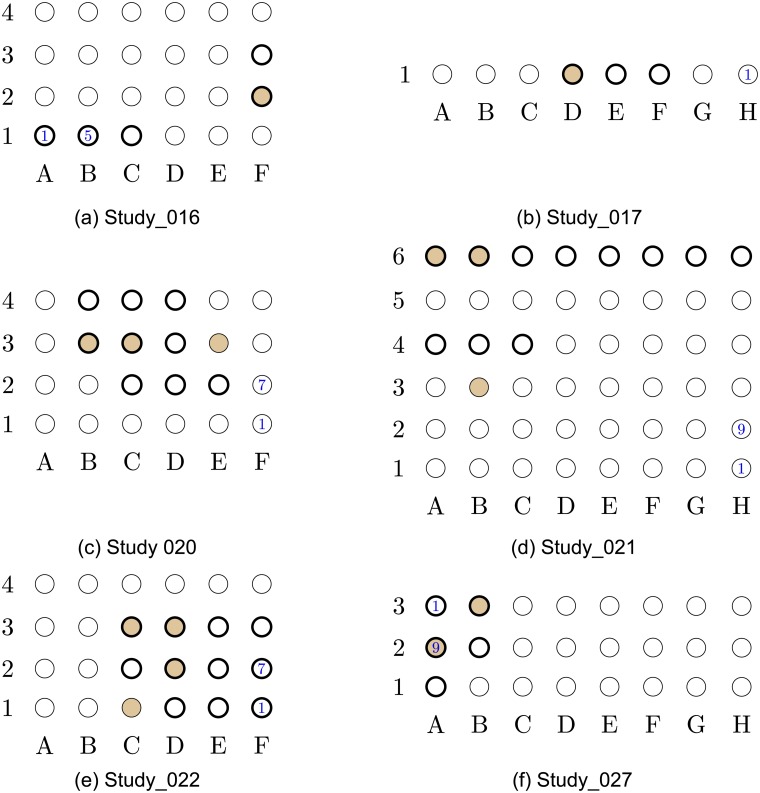
Localization results for data-sets Study_016 to Study_027. The EOI nodes are marked by a *bold annulus*, whereas the nodes detected by our proposed algorithm are marked by solid brown circles. (a) (*Top left*) Localization results for data-set Study_016. This is a successful localization, with *V*_p_ = 0.064. (b) (*Top right*) Localization results for data-set Study_017. This is a successful localization, with *V*_p_ = 0. (c) (*Middle left*) Localization results for data-set Study_020. This is a successful localization, with *V*_p_ = 0. (d) (*Middle right*) Localization results for data-set Study_021. This is a successful localization, with *V*_p_ = 0. (e) (*Bottom left*) Localization results for data-set Study_022. This is a successful localization, with *V*_p_ = 0. (f) (*Bottom right*) Localization results for data-set Study_027. This is a successful localization, with *V*_p_ = 0.

**Fig 3:**
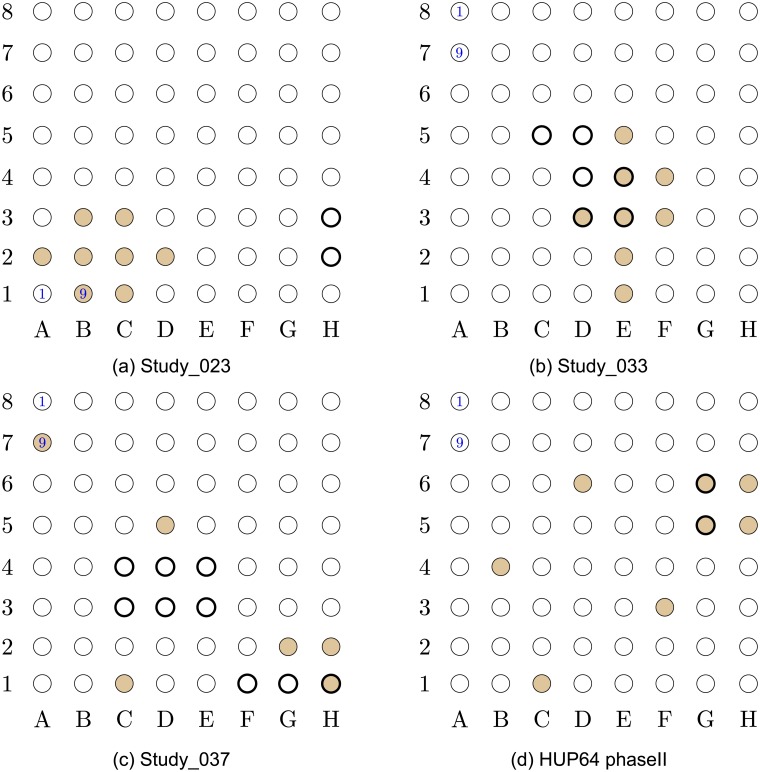
Localization results for data-sets Study_023 to HUP64_phaseII. The EOI nodes are marked by a *bold annulus*, whereas the nodes detected by our proposed algorithm are marked by solid brown circles. (a) (*Top left*) Localization results for data-set Study_023. This is a non-successful localization, with *V*_p_ = 0.143. (b) (*Top right*) Localization results for data-set Study_033. This is a successful localization, with *V*_p_ = 0.02. (c) (*Bottom left*) Localization results for data-set Study_037. This is a successful localization, with *V*_p_ = 0.051. (d) (*Bottom right*) Localization results for data-set HUP64_phaseII. This is a non-successful localization, with *V*_p_ = 0.071.

**Fig 4:**
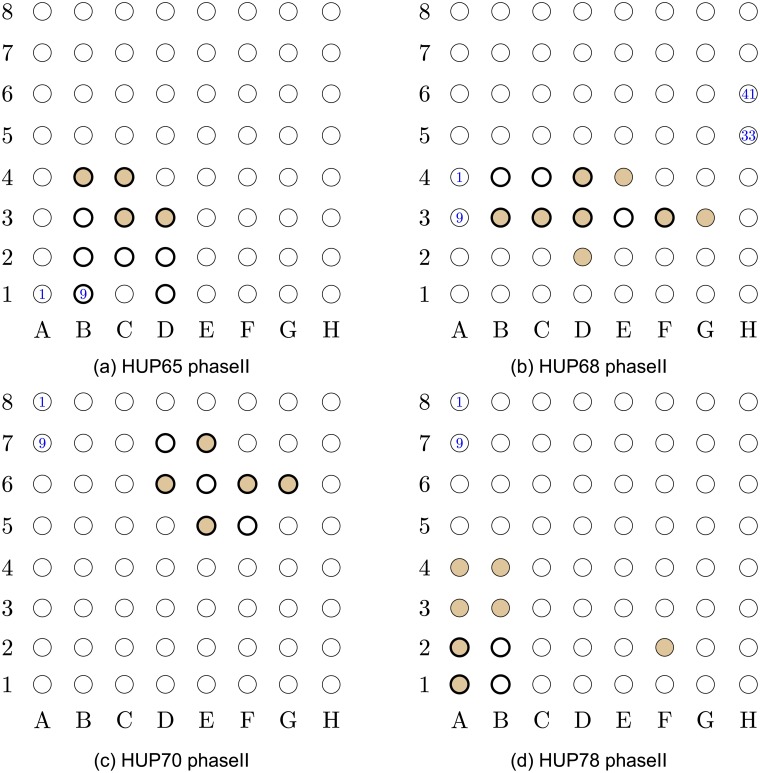
Localization results for data-sets HUP65_phaseII to HUP78_phaseII. The EOI nodes are marked by a *bold annulus*, whereas the nodes detected by our proposed algorithm are marked by solid brown circles. (a) (*Top left*) Localization results for data-set HUP65_phaseII. This is a successful localization, with *V*_p_ = 0. (b) (*Top right*) Localization results for data-set HUP65_phaseII. This is a successful localization, with *V*_p_ = 0. (c) (*Bottom left*) Localization results for data-set HUP70_phaseII. This is a successful localization, with *V*_p_ = 0. (d) (*Bottom right*) Localization results for data-set HUP78_phaseII. This is a successful localization, with *V*_p_ = 0.053.

**Fig 5:**
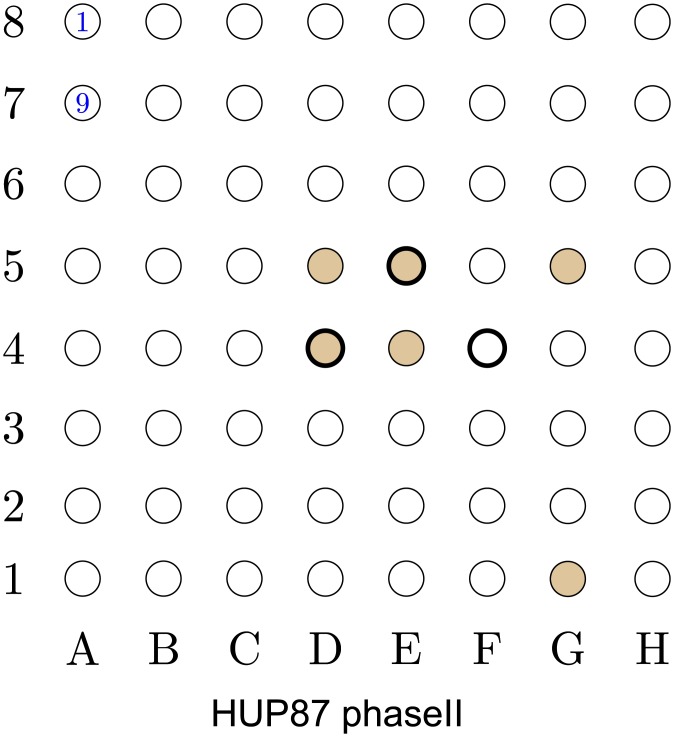
Localization results for data-set HUP87_phaseII. The EOI nodes are marked by a *bold annulus*, whereas the nodes detected by our proposed algorithm are marked by solid brown circles. This is a successful localization, with *V*_p_ = 0.037.

The localization results for the patients detailed in Table 1 are presented in Figs 1–5. As a ground truth we use the EOIs indicated by the neurologists and detailed in the data-sets reports. In Figs 1–5, the EOI electrodes (nodes) are marked by a *bold annulus*, whereas the nodes detected by our proposed algorithm are marked by solid brown circles. The reports in the iEEG portal contain a unique numbering for each electrode in each of the grids. This numbering is also included in the grids presented in Figs 1–5. For instance, in Fig 1-(a), node 1F is marked by 1 which corresponds to the numbering used in the report. This, together with the fact that node 2F is marked by 7, implies that node 3A corresponds to node 18 in the report.

The proposed algorithm applies a variant of PageRank on the estimated causal influence graph to calculate a rank (score) for each of the nodes. Then, natural candidates for the SOZ are the nodes with the top *p*_0_ percentile of scores. In order to verify that the calculated ranks are not due to chance and indeed capture an *evolving abnormal neural activity that leads to a seizure*, the proposed algorithm also calculates similar scores for an ensemble of recordings taken while the patient is *resting*. From this ensemble the algorithm creates an *empirical distribution* of the scores for each electrode, and requires electrodes in the SOZ to have a score in the top *p*_1_ percentile of the calculated empirical distribution. A detailed description of the inference procedure is provided in the Methods section.

The results in Figs 1–5 were obtained using *p*_0_ = 10 and *p*_1_ = 5. The values of *p*_0_ and *p*_1_ control the tradeoff between the *false positives* (identifying electrodes not in the SOZ) and the *false negatives* (SOZ electrodes not identified). Note that the number of indicated EOIs can be relatively large, for instance, in Fig 1-(b), 10 nodes out of 36 are indicated as EOIs. This number also differs between data-sets. The value of *p*_0_ was selected to provide a good balance between the success rate and the FPR, namely, inferring at most 10% of nodes in the grid as SOZ candidates. The value of *p*_1_ controls the significance level (enables the algorithm avoiding the possible bias caused by an inherent property of the patients’ brain). We discuss the implications of this parameter in the Discussion section (see the The structure of the estimated causal influence graph subsection and the A comparison with different inference approaches subsection).

Closely examining the localization maps in Figs 1–5, it can be observed that our algorithm successfully localized the SOZ (using the terminology defined above) in 17 out of the 19 data-sets. The two exceptions are data-set study_023 in Fig 3-(a) and data-set HUP64_phaseII in Fig 3-(d). Regarding data-set study_023 (Fig 3-(a)), it can be observed that the localization concentrates in the lower left corner of the grid. While the reports for this data-set clearly indicate that the SOZ is nodes 2H–3H (electrodes 58–59), they also state the following: “*The EEG showed fast activity at LTG #59 at 01:20:59, which then evolves into spike activity in LTG #58 and 59. At 01:21:10, there was spread of spike and wave activity to LTG #2, 3, 10, 11, 18, and 19*”. Thus, our algorithm accurately inferred the area to which the activity spread. By analyzing a time interval that significantly precedes the seizure start point marked in the reports (see the Methods section for a discussion regarding the analyzed time intervals), the inference can be significantly improved.

Regarding data-set HUP64_phaseII (Fig 3-(d)), it can be observed that four of the inferred nodes are concentrated around the EOI while the other four are spread over the grid. The reason for marking this inference as non-successful is the fact that *exactly* 50% of the electrodes overlap with the EOI, or with the nodes strictly adjacent to the EOI. Thus, this localization can be viewed as a *partial success*.

## Discussion

### The structure of the estimated causal influence graph

As mentioned above (see also the Methods section), the (statistical) significance of the calculated scores is evaluated in order to preclude scores which were obtained by chance or which are not a result of the evolving seizure activity. In other words, the objective of the post-processing is to verify that *the high scores are due to the evolving activity of an epileptic seizure* and *not an inherent property of the patients’ brain*. To test this hypothesis, the algorithm generates an empirical distribution of the scores calculated over *random* blocks recorded while the patient is resting, see the Methods section for a detailed description of this procedure. Our study shows that, for a specific patient, the estimated causal influence graph has patterns that are common between a rest state and the beginning of a seizure, namely, the beginning of the ictal state. This implies that one *must* account for rest blocks in order to avoid having the localization results *biased* by the inherent structure of the causal influence graph.

**Fig 6:**
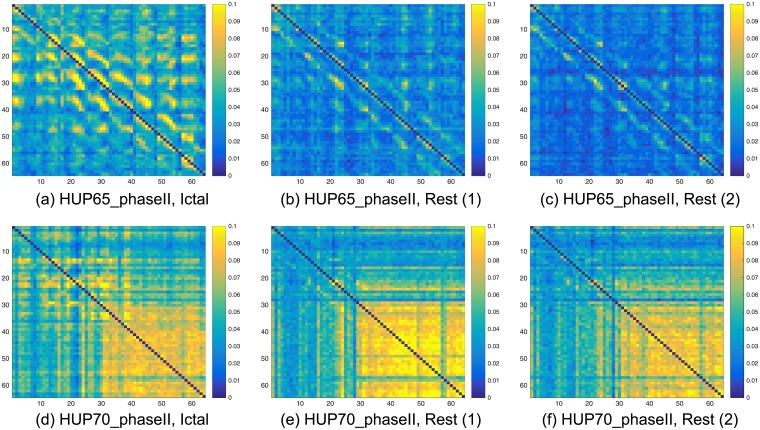
Heat maps illustrating the estimated causal influence graph for data-sets HUP65_phaseII and HUP70_phaseII. The left column corresponds to the first 10 seconds in the ictal blocks, while the two right columns correspond to two *random* 10 seconds rest blocks. (a)-(c) Heat maps of data set HUP65_phaseII. (d)-(f) Heat maps of data set HUP70_phaseII.

**Fig 7:**
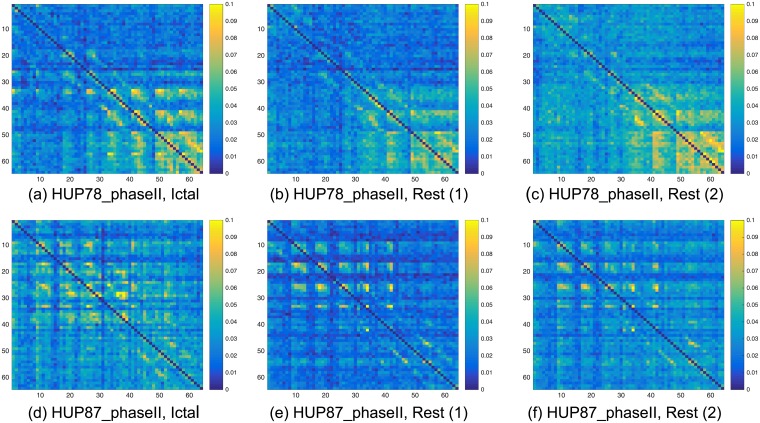
Heat maps illustrating the estimated causal influence graph for data-sets HUP78_phaseII and HUP87_phaseII. The left column corresponds to the first 10 seconds in the ictal blocks, while the two right columns correspond to two *random* 10 seconds rest blocks. (a)-(c) Heat maps of data set HUP78_phaseII. (d)-(f) Heat maps of data set HUP87_phaseII.

Figs 6 and 7 demonstrate that the causal influence graph estimated in rest blocks and in a block at the beginning of a seizure indeed have a common structure. Each of the sub-figures in Figs 6 and 7 is a heat map of an estimated graph (the entries are the estimations of the pair-wise causal influences). The procedure for creating (estimating) this graph is briefly discussed in the Methods section, while a detailed description is provided in S1 Text. The left column corresponds to the ictal blocks (beginning of a seizure), while the middle and right columns correspond to *random* blocks used as part of the post-processing procedure. Each row corresponds to a different data-set: HUP65_phaseII, HUP70_phaseII, HUP78_phaseII, and HUP87_phaseII. In each sub-figure, a yellow in the (*i*, *j*) location implies high estimated causal influence from node *i* to node *j* in the respective graph. The main dark blue diagonal in each of the heat maps corresponds to the causal influence between an electrode to itself that is set to zero.

It can be observed that, per data-set, i.e., in the same row in Fig 6 or in Fig 7, the heat maps follow a similar structure. On the other hand, this structure is different from one data-set to another (between different rows). In the first row of Fig 6, corresponding to data-set HUP65_phaseII, one can observe hot super and sub diagonals. In the second row of Fig 6, corresponding to HUP70_phaseII, one can observe a small hot region in the bottom right of the map. In the first row of Fig 7, corresponding to data-set HUP78_phaseII, one can also observe a hot region in the bottom right of the map, yet, this region is significantly smaller than the one in the second row of Fig 6. Finally, in the second row of Fig 7, that corresponds to HUP87_phaseII, one can observe small hot squares at the upper-left part of the map. These findings indicate that an inference procedure that ignores the structure during rest times, e.g., [16, 17], may not be aware of the structure that is present when there is no neural activity leading to a seizure. This may result in a *biased inference*.

One may conjecture that the structural resemblance demonstrated in Figs 6 and 7 is due to epileptic activity in a rest state, commonly referred to as interictal discharges [27, 28]. Yet, we note here that the starting point of the evaluated rest block is *randomly selected* (see the Methods section for details). Moreover, the patterns depicted in Figs 6 and 7 appear in *all* analyzed rest blocks. Thus, as interictal discharges are *relatively sparse*, we conjecture that this structure is *not due to the interictal discharges*. At the same time, we note that interictal discharges can be used to assist in localizing the SOZ [29]. Designing a robust method to incorporate the interictal discharges in our algorithm is part of our future research plans.

### A comparison with different inference approaches

A natural question is how good are the results reported in the Results section compared to the performance of other inference algorithms. To answer this question we tested two alternative inference approaches as well as two methods for estimating the pair-wise causal influences. Before discussing the alternative inference approaches we first provide some background on the problem of estimating the causal influence graph.

#### Estimating the causal influence graph

Recall that the weight of each edge in the graph quantifies the *causal influence* between a pair of recorded sequences. Generally speaking, causality measures can be divided into two groups: parametric and non-parametric, and can be estimated in the time domain or in the frequency domain. Parametric measures implicitly assume that the observed time-series follow a specific model (this makes estimation more efficient and simpler), however, a mismatch between the observed time-series and the assumed model usually leads to poor estimation results. Examples for parametric causality measures are *Granger causality* (GC) in the time domain [24], and its counterpart in the frequency domain, direct transfer function [30] (see [31] for a detailed review). As an accurate statistical model for ECoG recordings is not known, the proposed algorithm combines estimation of GC and the non-parametric causality measure of directed information (DI) [25], taken from the field of information theory [12]. The DI measure is closely related to the transfer entropy measure, see [32]. To estimate the pairwise DI, we use an estimator based on the *k*-nearest-neighbor (*k*-NN) principle [33], that extends the mutual information (MI) estimator derived in [34]. Our analysis indicates that this estimator is more accurate than other known non-parametric estimation approaches such as estimation of the causal conditional likelihood via kernel density estimation [16], or estimation via correlation integrals [17]. In the Methods section we briefly discuss the main ideas and techniques used to estimate the causal influence graph, a detailed description can be found in S1 Text.

As our algorithm combines both *k*-NN estimation of DI as well as estimation of GC, one may wonder about the inference performance when *only one of these methods is used*. Table 2 details a summary of the localization results when the causal influence graph is estimated using only GC *or* using only DI with *k*-NN estimation. The numbers of the inferred electrodes are provided in S2 Text. When obtaining the results specified in Table 2, the inference from the graph was based on PageRank followed by the post-processing step. The values of *p*_0_ and *p*_1_ were optimized for both *GC only* and *DI Only*, and in both cases the optimized values were very close to *p*_0_ = 10 and *p*_1_ = 5. It is clear that the proposed algorithm achieves significantly higher success rate while maintaining the same low average false positive detection rate.

**Table 2:** Summarized localization results for different graph estimation methods. GC refers to estimating the graph using *only* Granger causality, DI refers to estimating the graph using *only* *k*-NN DI estimation, and Proposed Algorithm refers to the algorithm proposed in the current paper. FPR refers to average false positive detection rate.

Method	Success Rate	FPR
GC only	0.63	0.039
DI only	0.737	0.03
**Proposed Algorithm**	0.895	0.03

#### Inferring the SOZ from the graph

We now consider alternative approaches to infer the SOZ from the estimated graph (the graph is estimated using the methodology of the proposed algorithm). The first approach is the net-flow inference [16, 17] where the rank of a node is the sum of the weights of its incoming edges subtracted from the sum of the weights of the outgoing edges. The second approach does not use post-processing and simply selects the SOZ to be the nodes with the highest scores (top 5%). This comparison indicates the effectiveness of the PageRank ranking approach as well as the importance of the post-processing step. These localization results are summarized in Table 3. The numbers of the inferred electrodes are provided in S2 Text. The superiority of the proposed algorithm relative to the alternative inference approaches can be clearly observed.

**Table 3:** Summarized localization results for different inference methods. Net-flow refers to the inference algorithm used in [16, 17]. Top 5% refers to choosing the nodes with the highest scores (top 5%). In both cases the graph is estimated using the methodology of the proposed algorithm. Proposed Algorithm refers to the algorithm proposed in the current paper. FPR refers to average false positive detection rate.

Method	Success Rate	FPR
Net-flow	0.684	0.053
Top 5%	0.789	0.035
**Proposed Algorithm**	0.895	0.03

### Seizure evolution through causal influence graphs

The proposed algorithm uses ECoG signals from two types of intervals (blocks): 10 seconds at the beginning of a seizure (an ictal block) and 10 seconds randomly selected from a period in which the patient is resting. Intuitively, in ictal blocks *the seizure activity has not spread out across the brain yet*, and therefore these blocks should give clear insights as to the SOZ location. The length of the analyzed blocks is chosen to be 10 seconds. This follows as these blocks are used to *estimate the weights in the causal-influence graph*, and this places two contradicting constraints on their length. On the one hand, the analyzed ECoG signals should be approximately stationary. According to [35], ECoG signals are approximately stationary only for a few seconds. On the other hand, the considered blocks should be long enough to facilitate non-parametric accurate estimation of the causal influence. Our study shows that blocks of 10 seconds provide a good tradeoff between the above two constraints (see the detailed discussion in the Description of the setup subsection).

**Fig 8:**
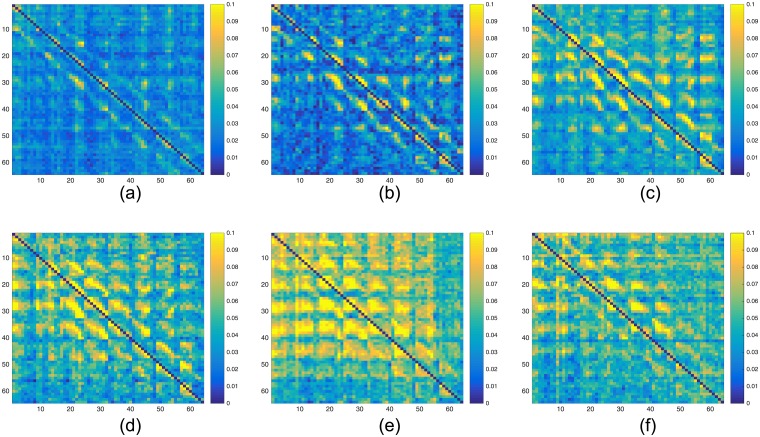
Heat maps illustrating the estimated causal influence graph for data-set HUP65_phaseII in different time intervals. (a) Heat map of the causal influence graph estimated from a rest block. (b) Heat map of the causal influence graph estimated from 10 seconds before a seizure (pre-ictal block). (c) Heat map of the causal influence graph estimated from a 10 seconds at the beginning of a seizure (ictal block). (d) Heat map of the causal influence graph estimated from 10-20 seconds *after* the beginning of a seizure. (e) Heat map of the causal influence graph estimated from 20-30 seconds *after* the beginning of a seizure. (f) Heat maps of the causal influence graph estimated from 30-40 seconds *after* the beginning of a seizure.

The heat maps in Figs 6 and 7 indicate that the causal influences in rest blocks (the middle and right column) are lower compared to those in the ictal blocks (depicted on the left column), namely, the graphs are more blue and less yellow. Extending this observation, our study shows that the seizure evolution process can be examined in terms of the causal influence graph, as depicted in Fig 8 for data-set HUP65 phaseII. Similarly to Figs 6 and 7, each of the sub-figures in Fig 8 is a heat map of an estimated graph (the entries are the estimations of the pair-wise causal influences), where in each sub-figure the graph was estimated from a different time window. The procedure for estimating these graphs is briefly discussed in the Methods section, while a detailed description is provided in S1 Text. It can be observed that in Fig 8-(a) (which corresponds to a rest state), the causal influence is relatively low (yet the pattern of hot super and sub diagonals is apparent). The causal influence is higher in Fig 8-(b) that shows the graph estimated from the recordings of pre-ictal state (10 seconds before the seizure starting point). The causal influence increases in Fig 8-(c)–8-(e), corresponding to the first 10 seconds (ictal block), 10 to 20 seconds after the seizure starts, and 20 to 30 seconds after the seizure starts, respectively. Finally, in Fig 8-(f), that corresponds to 30 to 40 seconds after the seizure starts, there is a decrease in the causal influence compared to Fig 8-(e). A possible explanation for this decrease is that after 30 seconds from the seizure starting point it already spread throughout the grid. Indeed, the reports corresponding to data-set HUP65_phaseII indicate that after about 30 seconds from the seizure starting point the activity was apparent in the whole grid: “*rhythmic sharps of variable amplitudes are recorded throughout the grid diffusely (generalized seizure electrographically)*”.

### High frequency oscillations

High frequency oscillations were recently suggested as good bio-markers for the epileptogenic zone [36–38]. Yet, as stated in [36], to record high frequency oscillations, the ECoG recordings must be sampled at a minimum rate of 2 KHz. The oscillatory events can then be visualized by applying a high-pass filter and increasing the time and amplitude scales. As 18 of the 19 data-sets were sampled at approximately 500 Hz, analysis of high frequency oscillations cannot be applied. Moreover, as discussed in S1 Text, to efficiently estimate the pair-wise causal influence graph we down-sample the recorded signals, see the discussion about the impact of the sampling rate on the signals memory order and the resulting number of samples required for accurate estimation. While the analyzed signals can represent any limited frequency band (not necessarily the low frequencies), the results presented in this work were obtained by analyzing the activity in frequencies below 100 Hz. We note that filtering out the high frequencies was also applied in [39].

### Incomplete representation of the causal influence network

On top of the sampling frequency limitations described in the previous section, it must be noted that the ECoG recordings in general, and the estimated causal-influence graph in particular, *do not* provide a complete representation of the epileptic network. Since it is not possible to record the electrical activity from the whole brain (the grids’ size is limited), the true SOZ may not be covered by the recording grid. In this work, we assume that the preceding analysis was executed, e.g., using EEG or MRI imaging, and that the grid was located based on a good (yet rough) estimation of the SOZ location. Another source of inaccuracy is the fact that the recorded signals might be influenced by (or correlated with) a strong signal originating from a location out of the grid. This may call for analysis of causal influence graphs in the presence of latent variables, see, for example, [40–42] and references therein. However, these works either assume a linear model, or derive estimation methods which require a very large number of samples. As discussed in S1 Text, the number of available samples for estimating the causal influences is inherently small, and thus these techniques cannot be used. Finally, as discussed above, since the electrodes in an ECoG grid are closely located, the recorded signals might be statistically dependent. In this case, to fully quantify the statistical causal influence between two electrodes, one must evaluate this influence when *statistically conditioning* on the rest of the electrodes. However, even for small grids, this task is too computationally demanding and requires a huge number of samples. Despite the incomplete representation of the causal-influence network via the pair-wise causal influence graph, the inference results presented above suggest that *the used approximation is accurate enough for the purpose of localizing the SOZ*.

### Computational aspects

A major concern regarding any automatic localization algorithm is the computational aspects [16, 17]. In particular, for large grids, the computational complexity of estimating the causal influence graph is high since *N*(*N* − 1) values must be estimated. This leads to the question: *can the proposed algorithm be executed in real-time to yield results within minutes from the time that the recording session ends?*

We assert that it can, given that the main computational load of our algorithm is the estimation of the causal-influence graphs of *the random rest blocks*, see the Methods section. This follows as the number of seizures per patient is relatively small (see Table 1 where data-set Study_033 is the largest with 17 seizures), while in order to create the empirical distributions we estimate the causal influence graph for 200 rest blocks. Note that there is no need to wait until the end of the recording session to execute this estimation task. In fact, estimation of the causal influence graphs for the rest blocks can be executed in parallel to the recording procedure, thus, significantly reducing the computational load at the end of the procedure. We further note that estimating the graph can be performed using a dedicated hardware (Graphics Processing Unit) and in parallel over several processors [43], reducing the required time even further. Finally, we emphasize that from the perspective of graph theory, the estimated graphs are very small (compared to graphs with thousands or even millions of nodes). Therefore, the computational complexity of the inference procedure based on the PageRank algorithm is negligible.

### The impact of the proposed algorithm and future research

The impact of an automatic localization algorithm could be significant, in particular in view of the improved inference performance reported in Tables 2 and 3. First, it can provide an objective point of view regarding the SOZ location. Second, the proposed algorithm can save analysis time for the neurologists by providing a pointer to a set of electrodes suspected to be located over the SOZ. Third, while the proposed algorithm focuses on inferring the SOZ, the techniques developed in this work can be used to learn other mechanisms and dynamics of the brain. For instance, understanding modifications in the neural network due to learning [44], or extending the above discussion on seizure evolution. Finally, we note that the proposed PageRank-based analysis of the graph can be applied after *any procedure for developing a weighted directed graph indicative of causal influences*. While due to computational complexity constraints, and the limited number of available samples, the proposed algorithm uses the pair-wise DI, in principle, any procedure that estimates a weighted directed graph (for instance, estimating the causally *conditioned* DI) can be used before applying the PageRank algorithm.

In terms of future research, we currently have three main directions: First, the proposed algorithm uses the rest periods to create the empirical distributions used in the post-processing stage. An interesting question is how to use these blocks to learn about the epileptic activity of the patient, thus improving the inference accuracy. We believe that by identifying rest blocks with interictal discharges, it will be possible to further take advantage of the recorded rest blocks. Second, the similarity between the structure of the graphs estimated in rest and pre-ictal blocks motivates analyzing these structures also during the seizure itself. Such an analysis can shed light on the transition from rest to seizure and on the propagation of the seizure activity over the network. Third, an important aspect in estimating the pair-wise causal influences (or any statistical functional that involves memory) is estimating the length of the auto-time-dependence of the ECoG recordings (for Gaussian signals this reduces to the actual length of the auto-correlation function). The parameter can also be interpreted as the Markov order of the sequence. Using tools from the theory of machine learning, namely, a data-driven estimator of the Markov order, in [45] we are studying the empirical distribution of the estimated Markov order over different states (rest and ictal) and different patients.

## Methods

### Ethics statement

The patients included in this study (listed in the iEEG portal [26]) provided a written and informed consent in accordance with the University of Pennsylvania Institutional Review Board and Mayo Clinic Institutional Review Board for inclusion in the current study.

### Description of the setup

We begin the Methods section with a formal description of the setup. In particular, we specify what parts of the ECoG recordings are analyzed by the algorithm, and formally define its output. In the subsequent subsections we discuss the application of the PageRank algorithm, and the method used to infer the SOZ.

The input to our algorithm are ECoG recordings from an epileptic patient (diagnosed to have a refractory epilepsy), as well as annotations information that indicates about the state of the patient in a given time interval (resting, pre-ictal, ictal, etc.). The labeling of these time intervals is done based on video recording of the patients. The annotations information also includes a report, composed by the expert neurologists, that specify the EOIs. The recordings and annotations information for all patients are listed in the International Epilepsy Electrophysiology (iEEG) portal [26], see Table 1 for the specific patient information. The objective of the algorithm is to localize the SOZ, namely, to find a (small) subset of electrodes that are located close to (above) the SOZ. We emphasize that the proposed algorithm *takes as input the time intervals of the seizures*, it *does not detect them*.

**Fig 9:**
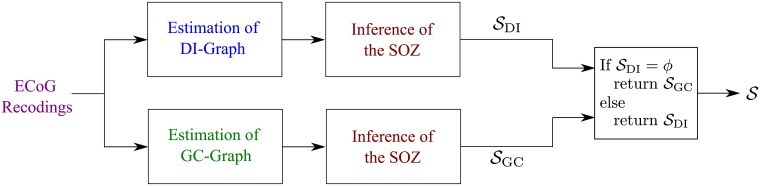
High-level block diagram of the proposed algorithm. $S_{\text{DI}}$ and $S_{\text{GC}}$ are the inferences (set of electrodes) from the DI-Graph and the GC-Graph, respectively; *ϕ* denotes the empty set; and S is the final set of inferred electrodes.

Fig 9 provides a high-level block diagram of the proposed algorithm. As discussed in the A comparison with different inference approaches subsection, the algorithm combines estimation of the causal influence graph quantified using the DI metric (DI-Graph), with estimation of the causal influence graph quantified using the GC measure (GC-Graph). Specifically, as the DI measure does not assume any parametric model for the data, the algorithm first infers the SOZ from the DI-Graph. While this works in most cases, it is possible that this inference will *not lead* to any candidate (electrode) to be part of the SOZ (this is discussed in the sequel). In this case, the algorithm infers the SOZ from the GC-Graph. As stated above, a detailed description of the procedure for estimating the DI-Graph and the GC-Graph is provided in S1 Text. The procedure for inferring the SOZ from the estimated graph is discussed below.

As indicated in Fig 9, the inputs to the proposed algorithm are the ECoG recordings. This leads to the following natural question: *which parts of the ECoG recordings are analyzed?* In contrast to [16] and [17] that focused only on the ECoG recordings corresponding to seizures, the proposed algorithm uses the annotations’ information and analyzes two types of time intervals (blocks):

1. A time interval at the *beginning* of the seizure, referred to as an **ictal block**. It is assumed that in these blocks *the seizure activity has not spread out across the brain yet*, and therefore these blocks should give a clear insight as to the SOZ location.
2. **Rest blocks** are *randomly sampled* from intervals that exclude seizures, artifacts, and blocks just before and after seizures. In this time, the patient is resting (awake).

**Fig 10:**
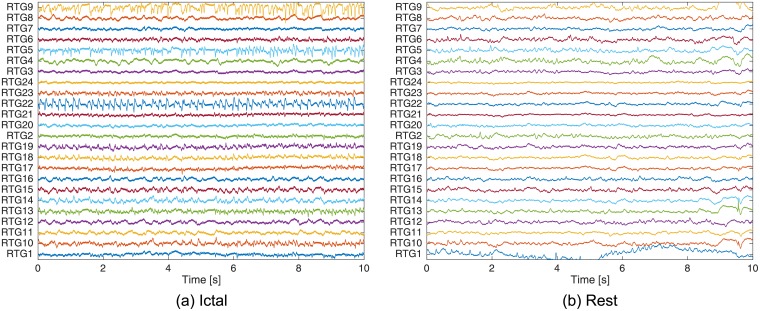
Exemplary recorded signals for data-set Study_016. The sampling rate is 500 Hz, while the block length is 10 seconds. (a) An ictal block. (b) A (randomly sampled) rest block.

The length of the analyzed blocks is chosen to be 10 seconds. An example of the recorded signals (ictal as well as rest blocks), for data-set Study_016, is depicted in Fig 10. The block length is chosen to be 10 seconds as these blocks are used to estimate the *pair-wise causal influences*, which places two contradicting constraints on the length of the analyzed blocks. On the one hand, as a statistical measure is estimated, the analyzed ECoG signals should be approximately stationary. According to [35], ECoG signals are approximately stationary only for a few seconds. On the other hand, the considered blocks should be long enough (contain enough samples) to facilitate accurate estimation of the statistical measure. Our study shows that blocks of 10 seconds provide a good tradeoff between the above two constraints. In particular, executing the proposed algorithm for several block lengths (5, 10, and 20 seconds) revealed that 10 seconds provides the best localization performance, as indicated in Table 4. A detailed description of the electrodes inferred to be part of the SOZ is given in S2 Text.

**Table 4:** Summarized localization results for different window lengths. FPR refers to average false positive detection rate.

Window length	Success Rate	FPR
5 seconds	0.579	0.03
20 seconds	0.631	0.03
**Proposed Algorithm (10 seconds)**	0.895	0.03

**Fig 11:**
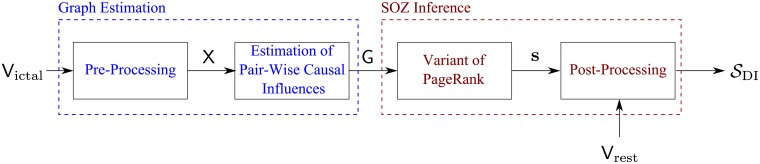
A block diagram of the procedure for calculating $S_{\text{DI}}$ (or $S_{\text{GC}}$). $V_{\text{ictal}}$ is a 10 ⋅ *F*_s_ × *N* matrix of the ECoG recordings; X is a matrix of the pre-processing output; G is the estimated causal-influence graph (of size *N* × *N*); **s** is the vector of scores generated by the (variant of the) PageRank ranking process; $V_{\text{rest}}$ is a 2000 ⋅ *F*_s_ × *N* matrix used to create the empirical distributions; and, S is a set of electrodes inferred to be the SOZ.

Fig 11 depicts a block diagram of the processing applied to estimate $S_{\text{DI}}$ (or $S_{\text{GC}}$). This processing consists of two main parts: estimation of the causal influence graph, and inference of $S_{\text{DI}}$ ($S_{\text{GC}}$) from the estimated graph. In this section we focus on the right part of Fig 11 (emphasized in dark red). Let the sampling rate in recording the ECoG signals be *F*_s_ Hz, and let the number of recorded electrodes be *N* (recall that typical values are *F*_s_ = 500 Hz and *N* = 64). The input to the first block in Fig 11 is a 10 ⋅ *F*_s_ × *N* matrix denoted by $V_{\text{ictal}}$. This matrix contains the recordings from the first 10 seconds of a seizure. The *i*^th^ column in $V_{\text{ictal}}$ corresponds to recordings from the *i*^th^ electrode. The output of this block is an *N* × *N* matrix G, representing a *complete directed graph* with *N* nodes, where the *i*^th^ node corresponds to the *i*^th^ recording electrode. The graph G does not contain self loops. The element in the *i*^th^ row and *j*^th^ column of (the matrix representation) G, ${\left[G\right]}_{i,j}$, is the weight of the edge between nodes *i* and *j*; it quantifies (via DI or GC) the causal influence of the signal recorded in the *i*^th^ electrode on the signal recorded in the *j*^th^ electrode. The values of $G_{i,i}$ are set to zero. A detailed description on how these quantities are estimated is provided in S1 Text.

To infer the nodes corresponding to the SOZ from the graph, we first note that a common problem in network analysis is to identify the most *important* nodes in the network. As the exact interpretation of importance is often *application dependent*, it can be quantified using many different measures [46]. In the current work we evaluate the importance of a node by quantifying the amount that this node serves as a *source of information flow*, i.e., causal influence, in the graph. The objective of the dark red part in Fig 11 (denoted by “SOZ Inference”) is to find these important nodes in G. A related problem is to *rank* the nodes in a network, and similarly to the case of importance, there are many definitions and algorithms for computing rankings [47]. One of these algorithms is the famous PageRank algorithm. Next, we discuss how the PageRank algorithm can be used to infer the SOZ from the graph G.

### Ranking the nodes in G via the PageRank algorithm

Recall our underlying hypothesis that the abnormal neural activity starts at the SOZ and then *spread* to the other electrodes. Hence, in terms of causal information flow, nodes in the SOZ should have *high outgoing flow* and *low incoming flow*. This reasoning led to the net-flow metric. The PageRank algorithm quantifies the importance of a node based on its *incoming* links (while accounting for the structure of the whole graph). A detailed description of the “vanilla” version of the PageRank algorithm (in the context of ranking web pages) is available in [48]. To quantify the importance of a node based on its *outgoing* links we propose to use the Reverse PageRank algorithm [22].

Using terminology taken from the hyperlink-induced topic search algorithm [21], PageRank assigns *authority* scores to the nodes. In a recursive manner, a high authority score is given to a node that is *linked by many other nodes with high authority scores*. Thus, the authority score can be seen as an in-flow score that *accounts for the structure in the graph*. To obtain an equivalent to the out-flow score, Reverse PageRank is used to calculate *hub* scores. Again, in a recursive manner, a high hub score is given to a node that is *linked to many other nodes with high hub scores*. Motivated by the arguments that led to the net-flow metric, we propose to use the difference between the hub and authority scores as the metric for ranking the nodes. Before formally describing how to calculate these metrics, we emphasize that in our algorithm the PageRank *does not model the propagation* of the seizure. Instead, it is used to *evaluate the importance* of a node in terms of its causal influence on the rest of the network.

To calculate the authority scores we apply a (modified) PageRank on the graph G (G can be either the DI-Graph or the GC-Graph). Let the matrix ${\bar{P}}_{j,i}$ be defined as:

$${\bar{P}}_{j,i}=\frac{G_{i,j}}{\sum_{k=1}^{N}G_{i,k}}.$$ (1)

Note that the elements of $\bar{P}$ are positive, while its *columns* sum to one. Therefore, the column vectors in $\bar{P}$ are in fact probability vectors. Further note that, in contrast to the PageRank described in [48] where the column vectors correspond to the uniform distribution, in (1) the elements in a given column of $\bar{P}$ can be different from each other. Next, the matrix P is generated from the matrix $\bar{P}$ by replacing any zero column in $\bar{P}$ with a vector containing entries that are all equal to $\frac{1}{N}$. Finally, the authorities (column) vector **a** is calculated as the solution of:

$$(\alpha P+\left(1-\alpha \right)v_{a}e^{T})a=a,$$ (2)

where **e** denotes the unity column vector, 0 < *α* < 1, and **v**_a_ is a column probability vector, i.e., a vector with positive elements that sum to unity. The vector **v**_a_ is commonly referred to as the *teleportation distribution*. The addition of this vector ensures that there is *always* a unique **a** that solves (2). Generally speaking, there are two main approaches for choosing **v**_a_. When **v**_a_ is close to uniform, it is common that PageRank is used to calculate a network *centrality measure*, thus calculating the importance of each node based on the structure of the entire graph. On the other hand, **v**_a_ can be a fixed *personalization* vector that is exploited to bias the result of the towards certain parts of the graph. This can be viewed as a *localized* measure of importance. The parameter *α*, commonly referred to as the *dumping factor*, controls the teleporation probability. Finally, the importance of a node is its corresponding probability in the vector **a**, which leads to the following two comments:

1. When solving (2) **a** is restricted to have a unit sum. Thus, letting I denote the identity matrix, solving (2) is equivalent to solving:
  $$\left(I-\alpha P\right)a=\left(1-\alpha \right)v_{\text{a}}.$$ (3)
2. The proposed algorithm uses a *soft personalization* vector **v**_a_ that weights the nodes according to their total incoming flow, namely:
  $$v_{\text{a},i}=\frac{\sum_{k=1}^{N}G_{k,i}}{\sum_{i=1}^{N}\sum_{j=1}^{N}G_{i,j}}.$$ (4)
  This approach biases the ranking procedure towards the initial guess which is based on the total inflow metric. Note that by choosing *α* ≈ 0, the obtained authority scores are approximately the normalized inflow. By using a larger value of *α*, the ranking further accounts for the structure of G. Following the detailed discussions in [20, 48], and based on the analyzed data sets, in this work we use *α* = 0.85.

As indicated above, to calculate the hub scores (that quantify the importance of a node based on its *outgoing* links) we propose to use the Reverse PageRank. This can be easily done by applying (1)–(4) with G replaced by its transpose $G^{T}$ and **v**_a_ replaced by **v**_h_, where the elements of **v**_h_ are calculated via:

$$v_{h,i}=\frac{\sum_{k=1}^{N}G_{i,k}}{\sum_{i=1}^{N}\sum_{j=1}^{N}G_{i,j}}.$$ (5)

The resulting vector of hub scores is denoted by **h**. Finally, the score of node *i* is given by:

$$s_{i}=h_{i}-a_{i}.$$ (6)

Thus, *s*_*i*_ quantifies the amount of total flow (of causal influence) for node *i*, *while accounting for the graph structure*.

### Post-processing, graph selection, and SOZ inference

Before discussing how to infer the SOZ, we note that it is common that neuronal activities associated with several seizures are recorded in each recording session (most data sets contain data associated with multiple seizures, see Table 1). Based on the assumption that there is a *single focus*, we combine these instances by *averaging* the estimated graphs (the DI-Graph or the GC-Graph). Slightly abusing the notation, this results in the graph G that is then used as the input to the PageRank algorithm. This approach was also taken in [16].

We now describe the method for selecting the set of nodes corresponding to the SOZ. Recall that the algorithms calculates both the DI-Graph and the GC-Graph. As there is no known statistical model for ECoG recordings, the algorithm first uses the DI-Graph for inferring the SOZ. As will be clear shortly, for some data-sets the inference based on the DI-Graph results in no candidate electrodes to be declared as part of the SOZ. In such a case the algorithm uses the GC-Graph to infer the SOZ.

Let $s={\left\{s_{i}\right\}}_{i=1}^{N}$ be the vector of scores calculated from the estimated DI-Graph, and let $S_{0}^{\left(\text{DI}\right)}\in \left\{1,2\ldots ,N\right\}$ be the set of nodes that constitute the top *p*_0_ percentile of **s**. Thus, $S_{0}^{\left(\text{DI}\right)}$ is a natural candidate to be declared as the SOZ. Yet, one better first verify that:

1. The calculated scores $S_{0}^{\left(\text{DI}\right)}$ are statistically significant, namely, they were not obtained by chance.
2. The calculated scores $S_{0}^{\left(\text{DI}\right)}$ are a *result of the evolving epileptic activity* and *not* an inherent property of the brain (see for example Figs 6 and 7).

A possible method to verify the above two points is via a comparison of the estimated scores *s*_*i*_ to *their* null-distribution. This null-distribution (specific for each score) should reflect the distribution of *s*_*i*_ when *there is no abnormal neural activity leading to a seizure*, such that a high *s*_*i*_ value will reflect a strong total flow *due to the abnormal activity (that leads to a seizure)*. As the true null-distributions are not known, we calculate an *empirical* distribution based on the recorded rest blocks. The rest blocks used to generate these empirical distributions are selected at random, and therefore, with high probability, satisfy the assumption that they do no include an evolving epileptic activity (see the discussion preceding Figs 6 and 7).

**Fig 12:**
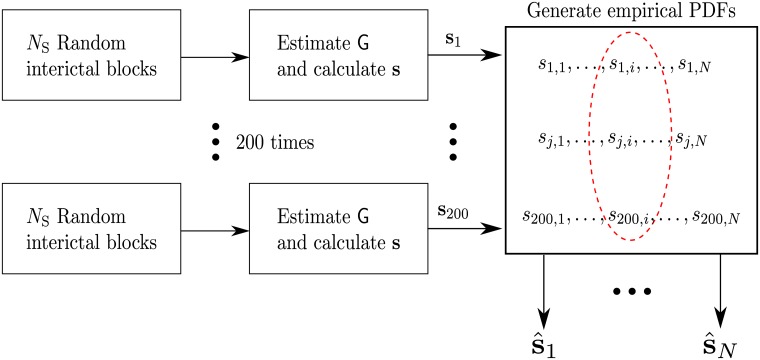
Ilustration of the the procedure for generating the empirical distributions for each node. ${\hat{s}}_{j},j=1,2,\ldots ,N$, denotes the empirical distribution of the *j*^th^ node.

The procedure for creating the empirical distributions is illustrated in Fig 12. Let *N*_S_ ≥ 1 denote the number of analyzed seizures (number of seizures in the data set). To create the empirical distributions we *randomly* choose *N*_S_ blocks (10 seconds time intervals) recorded while the patient is in a *rest* state. We emphasize that the starting point of these blocks is random, and the only constraint is that these blocks contain valid recordings (non-corrupted voltage traces). We apply the presented inference procedure (estimating the graphs, averaging, and calculating the sources scores) on the *N*_*S*_ blocks to obtain the scores ${\left\{\tilde{s_{i}}\right\}}_{i=1}^{N}$ that correspond to the currently sampled *random rest blocks*. By repeating this procedure 200 *independent* times we create an *empirical* null-distribution for each ${\tilde{s}}_{i}$. Note that an empirical distribution is generated for each *s*_*i*_, separately.

The algorithm now uses the generated empirical distributions as part of the SOZ inference. Let $S_{1}^{\left(\text{DI}\right)}\in \left\{1,2\ldots ,N\right\}$ denote the set of nodes for which *s*_*i*_ is in the top *p*_1_ percentile of the generated empirical distribution of ${\tilde{s}}_{i}$. Therefore, for a small value of *p*_1_, the scores of the nodes in the set $S_{1}^{\left(\text{DI}\right)}$ are significant. The algorithm now calculates the set $S_{\text{DI}}=S_{0}^{\left(\text{DI}\right)}\cap S_{1}^{\left(\text{DI}\right)}$, i.e., the set of nodes that have high scores for being sources in the graph, *and simultaneously* are statistically significant compared to their calculated empirical distributions. If this set is not empty, it is declared to be the SOZ. In case the set $S_{\text{DI}}$ is empty, the above procedure is repeated using the GC-Graph instead of the DI-Graph. The empirical distributions are generated by estimating the GC-Graph from the rest blocks, and the corresponding sets $S_{0}^{\left(\text{GC}\right)}$ and $S_{1}^{\left(\text{GC}\right)}$ are obtained. The SOZ is now declared to be $S_{\text{GC}}=S_{0}^{\left(\text{GC}\right)}\cap S_{1}^{\left(\text{GC}\right)}$. The algorithm terminates at this point even if the set $S_{\text{GC}}$ is empty.

The localization results detailed in the Results section were obtained using *p*_0_ = 10 and *p*_1_ = 5. In three out of the 19 data-sets detailed in Table 1 the set $S_{\text{DI}}$ was empty: Study_006, Study_021, and Study_033. In these data-sets, using the GC-Graph lead to a successful localization. Interestingly, in the two data-sets with non-successful localization (study_023 and HUP64_pahseII) the inference results based on the DI-Graph and the GC-Graph are very similar. Finally, we note that the results in Table 3, the “Top 5%” row, were obtained using *p*_0_ = 5 and *p*_1_ = 100 (no comparison to the empirical distributions).

## Supporting information

S1 Text Contains a detailed description of the methods for estimating the causal influence graphs. (PDF)

S2 Text Contains the numbers of inferred electrodes for all the discussed configurations. (PDF)

## References

[1] Fisher RS, v Emde Boas W, Blume W, Elger C, Genton P, Lee P, et al Epileptic seizures and epilepsy: definitions proposed by the International League Against Epilepsy (ILAE) and the International Bureau for Epilepsy (IBE). Epilepsia. 2005;46(4):470–472. doi: 10.1111/j.0013-9580.2005.66104.x 15816939 10.1111/j.0013-9580.2005.66104.x

[2] van Mierlo P, Papadopoulou M, Carrette E, Boon P, Vandenberghe S, Vonck K, et al Functional brain connectivity from EEG in epilepsy: Seizure prediction and epileptogenic focus localization. Progress in Neurobiology. 2014;121:19–35. doi: 10.1016/j.pneurobio.2014.06.004 25014528 10.1016/j.pneurobio.2014.06.004

[3] Yaffe RB, Borger P, Megevand P, Groppe AM, Kramer MA, Chu CJ, et al Physiology of functional and effective networks in epilepsy. Clinical Neurophysiology. 2015;126:227–236. doi: 10.1016/j.clinph.2014.09.009 25283711 10.1016/j.clinph.2014.09.009

[4] Duncan JS, Sander JW, Sisodiya SM, Walker MC. Adult epilepsy. The Lancet. 2007;367(9516):1087–1100. doi: 10.1016/S0140-6736(06)68477-8 10.1016/S0140-6736(06)68477-8 16581409

[5] Rosenow F, Luders H. Presurgical evaluation of epilepsy. Brain. 2001; p. 1683–1700. doi: 10.1093/brain/124.9.1683 11522572 10.1093/brain/124.9.1683

[6] Rummel C, Abela E, Andrzejak RG, Hauf M, Pollo C, Muller M, et al Resected brain tissue, seizure onset zone and quantitative EEG measures: Towards prediction of post-surgical seizure control. PLoS ONE. 2015;10(10):1–26. doi: 10.1371/journal.pone.0141023 10.1371/journal.pone.0141023 PMC4626164 26513359

[7] Brazier MAB. Spread of seizure discharges in epilepsy: Anatomical and electrophysiological considerations. Experimental Neurology,. 1972; p. 263–272. doi: 10.1016/0014-4886(72)90022-2 4559716 10.1016/0014-4886(72)90022-2

[8] Hill NJ, Gupta D, Brunner P, Gunduz A, Adamo MA, Ritaccio A, et al Recording human electrocorticographic (ECoG) signals for neuroscientific research and real-time functional cortical mapping. Journal of Visualized Experiments. 2012;64:1–5. 10.3791/3993 PMC3471287 22782131

[9] Kim S, Quinn CJ, Kiyavash N, Coleman TP. Dynamic and succinct statistical analysis of neuroscience data. Proceedings of the IEEE. 2014;102(5):683–698. doi: 10.1109/JPROC.2014.2307888

[10] Janzing D, Balduzzi D, Grosse-Wentrup M, Scholkopf B. Quantifying causal influences. The Annals of Statistics. 2013;41(5):2324–2358. doi: 10.1214/13-AOS1145

[11] Pearl J. Causality: Models, Reasoning, and Inference. Cambridge University Press, Cambridge; 2000.

[12] Cover TM, Thomas JA. Elements of Information Theory 2nd Edition. 2nd ed Wiley-Interscience; 2006.

[13] Quinn CJ, Kiyavash N, Coleman TP. Efficient methods to compute optimal tree approximations of directed information graphs. IEEE Transactions on Signal Processing. 2013;61(12):3173–3182. doi: 10.1109/TSP.2013.2259161

[14] Edmonds J. Optimum branchings. Journal of Research of the National Bureau of Standards.1967;71B(4):233–240. doi: 10.6028/jres.071B.032

[15] Soltani N. Inferring signaling structures in the brain via directed information. Doctoral Thesis, Stanford University; 2015.

[16] Malladi R, Kalamangalam G, Tandon N, Aazhang B. Identifying seizure onset zone from the causal connectivity inferred using directed information. IEEE Jour of Sel Topics in Sig Proc. 2016;10(7):1267–1283. doi: 10.1109/JSTSP.2016.2601485

[17] Sabesan S, Good LB, Tsakalis KS, Spanias A, Treiman DM, Iasemidis LD. Information flow and application to epileptogenic focus localization from intracranial EEG. IEEE Trans Neural Syst Rehabil Eng. 2009;17(3):244–253. doi: 10.1109/TNSRE.2009.2023291 19497831 10.1109/TNSRE.2009.2023291 PMC7841427

[18] Brin S, Page L. The anatomy of a large-scale hypertextual Web search engine. In: Computer Networks ISDN Systems; 1998. p. 107–117.

[19] Page L, Brin S, Motwani R, Winograd T. The PageRank Citation Ranking: Bringing Order to the Web. Stanford InfoLab; 1999.

[20] Franceschet M. PageRank: Standing on the shoulders of giants. Communications of the ACM. 2011;54(6):92–101. doi: 10.1145/1953122.1953146

[21] Kleinberg J. Authoritative sources in a hyperlinked environment. Journal of ACM. 1999;46:604–632. doi: 10.1145/324133.324140

[22] Bar-Yossef Z, Mashiach LT. Local approximation of PageRank and reverse PageRank. In: The 17th ACM conference on Information and knowledge management; 2008. p. 279–288.

[23] Zuo XN, Ehmke R, Mennes M, Imperati D, Castellanos FX, Sporns O, et al Network Centrality in the Human Functional Connectome. Cerebral Cortex. 2012;22:1862–1875. doi: 10.1093/cercor/bhr269 21968567 10.1093/cercor/bhr269

[24] Granger CWJ. Investigating causal relations by econometric models and cross-spectral methods. Econometrica. 1969;37(3):424–438. doi: 10.2307/1912791

[25] Jiao J, Permuter HH, Zhao L, Kim YH, Weissman T. Universal estimation of directed information. IEEE Transactions on Information Theory. 2013;59(10):6220–6242. doi: 10.1109/TIT.2013.2267934

[26] Wagenaar J, Brinkmann B, Ives Z, Worrell G, Litt B. A multimodal platform for cloud-based collaborative research. In: Proc. of Int. IEEE EMBS Conf. on Neur. Eng. San Diego, CA, USA; 2013. p. 1386–1389.

[27] Gelinas JN, Khodagholy D, Thesen T, Devinsky O, Buzsaki G. Interictal epileptiform discharges induce hippocampal–cortical coupling in temporal lobe epilepsy. Nature Medicine. 2016;22(6):641–650. doi: 10.1038/nm.4084 27111281 10.1038/nm.4084 PMC4899094

[28] Roopun AK, Simonotto JD, Pierce ML, Jenkins A, Nicholson C, Schofield IS, et al Interictal epileptiform discharges induce hippocampal–cortical coupling in temporal lobe epilepsy. Proceedings of the National Academy of Sciences. 2010;107(1):338–343.

[29] de Curtis M, Jefferys JGR, Avoli M. Interictal Epileptiform Discharges in Partial Epilepsy: Complex Neurobiological Mechanisms Based on Experimental and Clinical Evidence In: Noebels JL, Avoli M, Rogawski MA, Olsen RW, Delgado-Escueta AV, editors. Jasper’s Basic Mechanisms of the Epilepsies. 4th ed National Center for Biotechnology Information (US); 2012. 22787635

[30] Kaminski MJ, Blinowska KJ. A new method of the description of the information flow in the brain structures. Biological Cybernetics. 1991;65(3):203–210. doi: 10.1007/BF00198091 1912013 10.1007/BF00198091

[31] Blinowska KJ. Review of the methods of determination of directed connectivity from multichannel data. Medical & Biological Engineering & Computing. 2011;49(5):521–529. doi: 10.1007/s11517-011-0739-x 21298355 10.1007/s11517-011-0739-x PMC3097342

[32] Liu Y, Aviyente S. The relationship between transfer entropy and directed information. In: Proc. Stat. Sig. Proc. Workshop. Ann Arbor, MI, USA; 2012. p. 73–76.

[33] Vicente R, Wibral M, Lindner M, Pipa G. Transfer entropy—a model-free measure of connectivity for the neurosciences. Journal of Computational Neuroscience. 2011;30(1):45–67. doi: 10.1007/s10827-010-0262-3 20706781 10.1007/s10827-010-0262-3 PMC3040354

[34] Kraskov A, Stogbauer H, Grassberger P. Estimating mutual information. Physical Review E. 2004;69(6). doi: 10.1103/PhysRevE.69.066138 10.1103/PhysRevE.69.066138 15244698

[35] Kramer MA, Eden UT, Kolaczyk ED, Zepeda R, Eskandar EN, Cash SS. Coalescence and Fragmentation of Cortical Networks during Focal Seizures. Journal of Neuroscience. 2010;30(30):10076–10085. doi: 10.1523/JNEUROSCI.6309-09.2010 20668192 10.1523/JNEUROSCI.6309-09.2010 PMC2927849

[36] Zijlmans M, Jiruska P, Zelmann R, Leijten FSS, Jefferys JGR, Gotman J. High-frequency oscillations as a new biomarker in epilepsy. Annals of Neurology. 2012;71(2):169–178. doi: 10.1002/ana.22548 22367988 10.1002/ana.22548 PMC3754947

[37] others Z WJEM. High frequency oscillations and high frequency functional network characteristics in the intraoperative electrocorticogram in epilepsy. NeuroImage: Clinical. 2016;12:928–939. doi: 10.1016/j.nicl.2016.09.014 27882298 10.1016/j.nicl.2016.09.014 PMC5114532

[38] Gliske SV, Irwin ZT, Chestek C, Stacey WC. Effect of sampling rate and filter settings on High Frequency Oscillation detections. Clinical Neurophysiology. 2016;127:3042–3050. doi: 10.1016/j.clinph.2016.06.029 27472539 10.1016/j.clinph.2016.06.029 PMC4980189

[39] Khambhati AN, Davis KA, Oommen BS, Chen SH, Lucas TH, Litt B, et al Dynamic network drivers of seizure generation, propagation and termination in human neocortical epilepsy. PLoS Comput Biol. 2015;11(12):1–19. doi: 10.1371/journal.pcbi.1004608 10.1371/journal.pcbi.1004608 PMC4682976 26680762

[40] Hosseini H, Kannan S, Zhang B, Poovendran R. Learning temporal dependence from time-series data with latent variables. In: IEEE International Conference on Data Science and Advanced Analytics. Montreal, Canada; 2016.

[41] Rahimzamani A, Kannan S. Network inference using directed information: The deterministic limit. In: 54th Annual Allerton Conference on Communication, Control, and Computing. Monticello, IL,USA; 2016.

[42] Salehkaleybar S, Etesami J, Kiyavash N. Identifying nonlinear 1-Step causal influences in presence of latent variables. https://arxivorg/abs/170106605. 2016;.

[43] Wollstadt P, Martinez-Zarzuela M, Vicente R, Diaz-Pernas FJ, Wibral M. Efficient transfer entropy analysis of non-stationary neural time series. PLoS One. 2014;9(7):1–22. doi: 10.1371/journal.pone.0102833 10.1371/journal.pone.0102833 PMC4113280 25068489

[44] Zhiting C, Neveu CL, Byrne JH, Aazhan B. On inferring functional connectivity with directed information in neuronal networks. In: Asilomar Conference on Signals, Systems and Computers. Pacific Grove, CA, USA; 2016.

[45] Murin Y, Goldsmith A, Aazhang B. Estimating the memory order of electrocorticography recordings. Submitted to IEEE Transactions on Biomedical Engineering. 2017;. 10.1109/TBME.2019.2896076 30714907

[46] Brandes U, Erlebach T (Eds). Network Analysis: Methodological Foundations, Lecture Notes in Computer Science. vol. 3418 Springer-Verlag; 2005.

[47] Langville AN, Meyer CD. Who’s #1?: The Science of Rating and Ranking. Princeton University Press; 2012.

[48] Gleich DF. PageRank Beyond the Web. SIAM Review. 2015;57(3):321–363. doi: 10.1137/140976649

