# Supplementary material for: SozRank: A new approach for localizing the epileptic seizure onset zone
Source: PLoS Comput Biol. 2018 Jan 30;14(1):e1005953. doi: 10.1371/journal.pcbi.1005953 (PMC5806930; doi:10.1371/journal.pcbi.1005953)
Supplement: S1 Text — (PDF) [file pcbi.1005953.s001.pdf]

S1 Text - Supporting Information for “SozRank: A new approach for localizing the epileptic seizure onset zone”

Yonathan Murin[1], Jeremy Kim[1], Josef Parvizi[2], Andrea Goldsmith[1]

[1] Department of Electrical Engineering, Stanford University, Stanford, CA, USA
[2] Department of Neurology & Neurological Sciences, Stanford University, Stanford, CA, USA

1 Introduction

This document describes the estimation of the causal influence graph G from the ECoG recordings. Let the sampling rate in recording the ECoG signals be F_s Hz, and let the number of recorded electrodes be N (recall that typical values are $F_s = 500$ Hz and $N = 64$). A block diagram of this estimation procedure is depicted in Figure 1. The input to the first block in Figure 1 is a $10 \cdot F_s \times N$ matrix V. The ith column in V corresponds to recordings from the ith electrode. The output of the right block in Figure 1 is an $N \times N$ matrix G, representing a *complete directed graph* with N nodes, where the ith node corresponds to the ith recording electrode. The graph G does not contain self loops. The element in the ith row and j^{th} column of (the matrix representation) G, $[\mathsf{G}]_{i,j}$, is the weight of the edge between nodes i and j; it quantifies (via directed information (DI) or Granger causality (GC)) the causal influence of the signal recorded in the ith electrode on the signal recorded in the jth electrode. The values of $\mathsf{G}_{i,i}$ are set to zero.

As indicated by Figure 1, the estimation procedure consists of two steps: pre-processing and estimation of the pair-wise causal influences. The objective of the pre-processing step is to prepare the raw data for the estimation procedure. We begin with a brief description of the DI functional that motivates the specific design of the pre-processing phase.

2 Directed Information - Notations, Definitions and Background

We first formally introduce the following notations. We denote random variables (RVs) by upper case letters, X, and their realizations with the corresponding lower case letters. We use the short-hand notation X_1^N to denote the sequence $\{X_1, X_2, \ldots, X_N\}$. We denote random processes using boldface letters, e.g., $\mathbf{X}$. Matrices are denoted by sans-serif font, e.g., V. We denote sets by calligraphic letters, e.g., $\mathcal{S}$, where $\mathcal{R}$ denotes the set of real numbers. $f_X(x)$ denotes the probability density function (PDF) of a continuous RV X on $\mathcal{R}$, $\log(\cdot)$ denotes the natural basis logarithm, and $\mathbb{E}\{\cdot\}$ denotes expectation.

Recall the definitions of *differential* entropy and mutual information (MI) from [1, Ch. 8]. Let $X \in \mathcal{X} = \mathcal{R}^{d_x}$ and $Y \in \mathcal{Y} = \mathcal{R}^{d_y}$ be RVs with marginal PDFs $f_X(x)$ and $f_Y(y)$, respectively, and joint PDF $f_{X,Y}(x,y)$. The *differential* entropy of X is defined as:

$$h(X) \triangleq -\mathbb{E}\{\log f_X(x)\} = -\int_{\mathcal{X}} f_X(x) \log f_X(x) dx, \tag{1}$$

where the differential entropy of Y, $h(Y)$, is defined similarly. The MI between X and Y is given by:

$$I(X;Y) \triangleq \int_{\mathcal{X}} \int_{\mathcal{Y}} f_{X,Y}(x,y) \log \frac{f_{X,Y}(x,y)}{f_X(x) f_Y(y)} dx dy, \tag{2}$$

and therefore can also be written as $I(X;Y) = h(Y) - h(Y|X) = h(X) + h(Y) - h(X,Y)$. The mutual information between the *sequences* X_1^N and Y_1^N is similarly given by $I(X_1^N; Y_1^N)$.

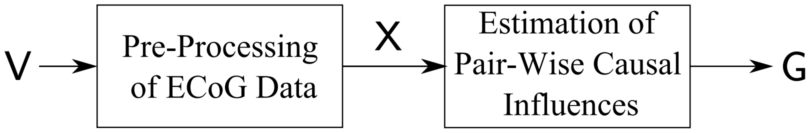

Figure 1: **A Block diagram of the procedure for estimating the causal influence graph** G. V is a $10 \cdot F_s \times N$ matrix of the ECoG recordings; X is a matrix of the pre-processing output; G is the estimated causal-influence graph (of size $N \times N$).

Let $\mathbf{X}$ and $\mathbf{Y}$ be arbitrary discrete-time continuous-amplitude random processes, and let $X^N \in \mathcal{R}^N$ and $Y^N \in \mathcal{R}^N$, be N-length sequences. Using the chain rule for MI [1, Ch. 2.5], the MI between X^N and Y^N can be written as:

$$I(X^N;Y^N) = \sum_{i=1}^{N} I(X_1^N;Y_i|Y_1^{i-1}). \tag{3}$$

Differently from MI, the DI from X^N to Y^N is defined as [2, *eq.* (1)]:

$$I(X^N \to Y^N) \triangleq \sum_{i=1}^{N} I(X_1^i;Y_i|Y_1^{i-1}). \tag{4}$$

Thus, in contrast to MI which quantifies a *measure of dependence* between two sequences, DI aims at quantifying the *causal influence* of the sequence X^N on the sequence Y^N (note that in the first term in (4) is X_1^i, while in (3) it is X_1^N). It can be observed that $I(X^N \to Y^N)$ depends on the sequence length N, thus, it is more insightful to consider the *DI rate* between the processes $\mathbf{X}$ and $\mathbf{Y}$, which is defined as [2, *eq.* (12)]:

$$I(\mathbf{X} \to \mathbf{Y}) \triangleq \lim_{N\to\infty} \frac{1}{N} I(X^N \to Y^N), \tag{5}$$

provided that this limit exists. We now make the following assumptions regarding the processes $\mathbf{X}$ and $\mathbf{Y}$.

A1) The random processes $\mathbf{X}$ and $\mathbf{Y}$ are *assumed to be stationary, ergodic, and Markovian of order* M in the observed sequences. The *stationarity* assumption implies that the statistics of the considered random processes are constant throughout the observed sequences. From a practical perspective, stationarity is required to ensure that the causal influence does not change over the observed sequences. Thus, the block length is chosen such that the analyzed signals are (approximately) stationary. *Ergodicity* is assumed to ensure that the observed sequences truly represent the underlying processes. Finally, the *Markovity* assumption is common in modeling *real-life systems which have finite memory*, in particular in neuroscience [3, 4, 5]. We formulate the assumption of Markovity of order m in the observed sequences via:

$$f(y_i|Y_1^{i-1}) = f(y_i|Y_{i-m}^{i-1}) \text{ and } f(y_i|Y_1^{i-1}, X_1^i) = f(y_i|Y_{i-m}^{i-1}, X_{i-m}^{i-1}), i > m.$$

Note that here we use the simplifying assumption that the dependence of y_i on past samples of Y_1^{i-1} and X_1^i is of the same order m. Further note that in the above Markovity assumption, we implicitly assume that given $(Y_{i-m}^{i-1}, X_{i-m}^{i-1})$, y_i is independent of X_i, which reflects a setting where X_i and Y_i are simultaneously measured, as in the case of ECoG recording.

A2) The entropy of the first sample y_1 exists: $|H(Y_1)| < \infty$.

A3) The following holds: $|H(Y_{m+1}|Y_1^m, X_1^m)| < \infty$.

In the context of the current work, Assumptions **A2**) and **A3**) are required to mathematically insure that the DI rate exists and is equal to a simple expression which depends on the finite memory

length m. Moreover, Assumptions **A2**) and **A3**) prevent the degenerate case of deterministic Y_1 or deterministic relationship between Y_{m+1} and Y_1^m, X_1^m. It is reasonable to assume that these conditions hold for the ECoG recordings which always contain some level of randomness. Under Assumptions **A1**)–**A3**), [5, Lemmas 3.1 and 3.2] imply that $I(\mathbf{X} \to \mathbf{Y})$ exists and is equal to:

$$I(\mathbf{X} \to \mathbf{Y}) = I(X_{i-m}^{i-1}; Y_i | Y_{i-m}^{i-1}), \quad i > m. \tag{6}$$

In view of (6), $I(\mathbf{X} \to \mathbf{Y})$ may have the following interpretation:

Given the past of the sequence Y, how much does the past of the sequence X helps in predicting the next sample of Y.

One can observe that this interpretation of the DI rate is identical to the interpretation of GC [6]. Yet, while GC is a parametric measure, DI does not assume any underlying statistical model. Next, we discuss the pre-processing of the ECoG data.

3 Pre-Processing

The pre-processing consists of 4 steps:

1. First, the common reference is removed from all the recorded signals [7, 8].
2. Then, each column of the matrix V is filtered using a 60 Hz notch filter to remove the line-noise [8, 9].
3. Next, each column of the matrix V is down-sampled to 100 Hz (recall that the sampling rate in the iEEG portal is between 500 Hz and 5 KHz). Since each block is 10 seconds long, this implies that the size of the matrix X in Figure 1 is $1000 \times N$.
4. Finally the mean of each column is removed and each column is normalized to have a unit variance.

To understand the reasoning behind down-sampling, we note that to accurately estimate the causal influence two contradicting constraints should be satisfied. On one hand, the sequence from which the causal influence is estimated should be approximately stationary (see Assumption **A1**)). According to [7], ECoG signals are approximately stationary only for a few seconds. Thus, the number of samples that can be used for estimation is limited. On the other hand, as stated in [10, Sec. V.E], the number of samples required for accurate estimation grows *exponentially* with the Markov order m (this follows as increasing m can be viewed as increasing the state space). Thus, if m is too large, one cannot hope to accurately estimate the causal influence. Now, the Markov order m should reflect the *auto-time-dependence* of the ECoG signals (auto-correlation in the case of Gaussian signals), namely, for a fixed auto-time-dependence τ (in milliseconds), m should satisfy $\tau = \frac{m}{F_s}$. Hence, for a given τ (which is a property of the considered continuous signal), increasing F_s requires also increasing m. However, the value of m must be controlled due to the limited number of samples. A possible approach to control the value of m, such that it will capture auto-time-dependence τ, is to reduce F_s via down-sampling.

The work [5] roughly estimated the Markov order m to be at the order of tens of milliseconds. For such values, when $F_s = 500$ Hz, one must use $m > 10$. At the same time, the number of ECoG samples one observes in a 10 seconds block (recall that the block cannot be too long due to the stationarity requirement), is far from sufficient for accurate estimation (when $m > 10$). To tackle this challenge we chose to *down-sample* the ECoG signals to 100 Hz, thus, the required m is significantly smaller, i.e., smaller than 10, and this can enable an accurate estimation. Note that the above down-sampling filters out all data in frequencies larger than 100 Hz. Filtering the high frequencies was also applied in [8].

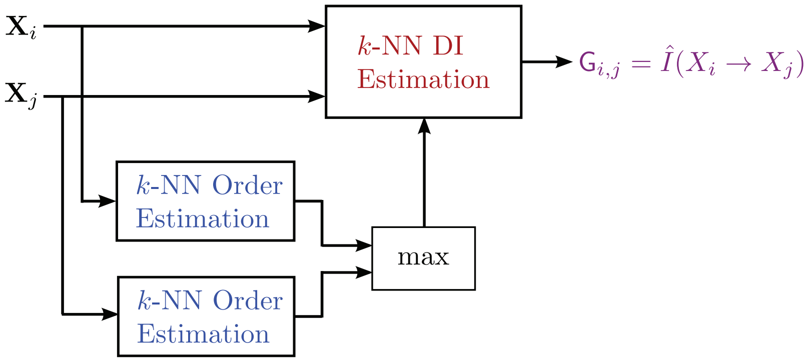

Figure 2: **A Block diagram of the procedure for estimating the pair-wise directed information**. $\mathbf{X}_i$ and $\mathbf{X}_j$ are columns in the matrix X. The result is the stored in $\mathsf{G}_{i,j}$.

To conclude this discussion we note that [5] proposed a different method to deal with the challenge of high values of m. Using the terminology in (6), [5] proposed the following approximation:

$$
\begin{aligned}
I(\mathbf{X} \to \mathbf{Y}) &= I(X_{i-m}^{i-1}; Y_i | Y_{i-m}^{i-1}) \\
&= I(X_{i-m}, X_{i-m+1}, ..., X_{i-1}; Y_i | Y_{i-m}, Y_{i-m+1}, ..., Y_{i-1}) && (7) \\
&\approx I(X_{i-m}, X_{i-m+\Delta}, X_{i-m+2\Delta}, ..., X_{i-1}; Y_i | Y_{i-m}, Y_{i-m+\Delta}, Y_{i-m+2\Delta}, ..., Y_{i-1}), && (8)
\end{aligned}
$$

where $1 \leq \Delta \leq m$ is an integer. We emphasize that this step was used *only* in estimating the causal influence. While this indeed reduces the required number of samples for estimation, it is not clear how Δ should be chosen. Moreover, the impact of the samples omitted when approximating (7) by (8) is not clear. In view of the common approach to analyze frequencies lower than 100 Hz [8], we believe that down-sampling is more robust and leads to better results as it accounts for all the information in the low frequencies. On the other hand, the approach of (8) ignores the dependence on the samples $X_{i-m+1}, X_{i-m+2}, \ldots, X_{i-m+\Delta-1}, X_{i-m+\Delta+1}, \ldots$ and $Y_{i-m+1}, Y_{i-m+2}, \ldots, Y_{i-m+\Delta-1}, Y_{i-m+\Delta+1}, \ldots$.

4 Estimating the Pair-Wise Causal Influences

A block diagram of the procedure for estimating the DI between the signal recorded in the ith electrode and the the signal recorded in the jth electrode is depicted in Figure 2.

From (6) it can be observed that the DI, and therefore its estimation, are functions of the Markov order m. Clearly, this value is not known apriori. Hence, m is first estimated, and then, its estimation is used in estimating the DI functional. To estimate m we use a locally weighted prediction calculated from the 8-nearest-neighbors (NN) of each sample tuple, thus, extending the approach of [11]. We note here that in order to maintain a reasonable computational complexity we estimate an m value for each time-series, i.e., column of X. Then, when estimating the DI, we use the value $\max\{m_i, m_j\}$, see Figure 6.

Given an estimation of m, the DI is estimated using the k-NN approach with $k = 5$. We use the estimator presented in [12] which extends the MI estimator derived in [13]. A detailed analysis of the procedure for estimating the pair-wise DI is out of the scope of this document. We refer the reader to [14, 15, 16] for a detailed description and discussion of the estimation method and its properties.

Recall that in addition to estimating the DI, the proposed algorithm also estimates the GC measure. This is done by using the Multi-Variate Granger Causality (MVGC) toolbox [17], as discussed in [9].

5 Generating the Causal Influence Graph

The graph G is constructed by defining each node to correspond to a recording electrode and each weight of an edge to quantify the respective causal influence. More precisely, let $[\mathsf{X}]_{[:,i]}$ denote the ith column of the matrix X. Then, the weight of the edge between the ith node and the jth node in G is given by:

$$[\mathsf{G}]_{i,j} = \hat{I}([\mathsf{X}]_{[:,i]} \rightarrow [\mathsf{X}]_{[:,j]}), \quad [\mathsf{G}]_{i,i} = 0, \tag{9}$$

where $\hat{I}(\mathbf{X} \rightarrow \mathbf{Y})$ denotes the estimation of DI (or GC). Note that the diagonal elements of G are set to zero, namely, there is no causal influence from a signal to itself.

We conclude this supplementary document with the observation that as $[\mathsf{G}]_{i,j}$ is *estimated* from a finite number of samples, one may want to assess the statistical significance of this estimation. A lack of statistical significance may imply that there is no causal influence between the considered signals, and the estimated values are due to either noise or due to estimation error. In such a case one may choose to set $[\mathsf{G}]_{i,j} = 0$. While for estimating GC there are known methods for quantifying the statistical significance [17, Sec. 2.5], such a method is not known for the problem of estimating DI (when the time-series samples are taken from a discrete alphabet [18] derived an asymptotically optimal null-distribution of the DI). An alternative method for evaluating the statistical significance is via a non-parametric bootstrapping procedure in the spirit of [19]. The main drawback of such a procedure is the tremendous increase in computational complexity, since applying such a bootstrapping procedure amounts to multiplying the computational complexity by a factor of at least 20 (note that the estimation of the DI values constitute the main computational load of the algorithm). For this reason, the statistical significance of a given pair-wise estimation (for the case of DI) is not evaluated. Instead, the algorithm uses the post-processing phase, as discussed in the Methods section in the main manuscript.

References

[1] T. M. Cover and J. A. Thomas, *Elements of Information Theory 2nd Edition*, 2nd ed. Wiley-Interscience, 2006.

[2] J. Jiao, H. H. Permuter, L. Zhao, Y.-H. Kim, and T. Weissman, "Universal estimation of directed information," *IEEE Transactions on Information Theory*, vol. 59, no. 10, pp. 6220–6242, Oct. 2013.

[3] D. Lederman and J. Tabrikian, "Classification of multichannel eeg patterns using parallel hidden markov models," *Medical & Biological Engineering & Computing*, vol. 50, no. 4, pp. 319–328, Apr. 2012.

[4] T. Wissel, T. Pfeiffer, R. Frysch, R. T. Knight, E. F. Chang, H. Hinrichs, J. W. Rieger, , and G. Rose, "Hidden markov model and support vector machine based decoding of finger movements using electrocorticography," *Journal of Neural Engineering*, vol. 10, no. 5, Oct. 2013.

[5] R. Malladi, G. Kalamangalam, N. Tandon, and B. Aazhang, "Identifying seizure onset zone from the causal connectivity inferred using directed information," *IEEE Jour. of Sel. Topics in Sig. Proc.*, vol. 10, no. 7, pp. 1267–1283, 2016.

[6] C. W. J. Granger, "Investigating causal relations by econometric models and cross-spectral methods," *Econometrica*, vol. 37, no. 3, pp. 424–438, 1969.

[7] M. A. Kramer, U. T. Eden, E. D. Kolaczyk, R. Zepeda, E. N. Eskandar, and S. S. Cash, "Coalescence and fragmentation of cortical networks during focal seizures," *Journal of Neuroscience*, vol. 30, no. 30, pp. 10 076–10 085, 2010.

[8] A. N. Khambhati, K. A. Davis, B. S. Oommen, S. H. Chen, T. H. Lucas, B. Litt, and D. S. Bassett, “Dynamic network drivers of seizure generation, propagation and termination in human neocortical epilepsy,” *PLoS Comput. Biol.*, vol. 11, no. 12, pp. 1–19, 2015.

[9] N. Soltani, *Inferring signaling structures in the brain via directed information*. Doctoral Thesis, Stanford University, 2015.

[10] J. Jiao, K. Venkat, Y. Han, and T. Weissman, “Minimax estimation of functionals of discrete distributions,” *IEEE Transactions on Information Theory*, vol. 61, no. 5, pp. 2835–2885, May 2015.

[11] M. Ragwtiz and H. Kantz, “Markov models from data by simple nonlinear time series predictors in delay embedding spaces,” *Physical Review E*, vol. 65, no. 5, pp. 1–12, 2002.

[12] R. Vicente, M. Wibral, M. Lindner, and G. Pipa, “Transfer entropy - a model-free measure of connectivity for the neurosciences,” *Journal of Computational Neuroscience*, vol. 30, no. 1, pp. 45–67, 2011.

[13] A. Kraskov, H. Stogbauer, and P. Grassberger, “Estimating mutual information,” *Physical Review E*, vol. 69, no. 6, 2004.

[14] M. Wibral, R. Vicente, and M. Lindner, “Transfer entropy in neuroscience,” *Directed Information Measures in Neuroscience, Understanding Complex Systems*, pp. 3–36, 2014.

[15] Y. Murin, “On k-nn estimation of directed information,” Tech. Rep., 2017, available at *https://arxiv.org/abs/1711.08516.*

[16] Y. Murin, J. Kim, and A. Goldsmith, “Tracking epileptic seizure activity via information theoretic graphs,” in *Asilomar Conference on Signals, Systems and Computers*, Pacific Grove, CA, USA, 2016.

[17] L. Barnett and A. Sheth, “The mvgc multivariate granger causality toolbox: A new approach to granger-causal inference,” *Journal of Neuroscience Methods*, vol. 223, pp. 50–68, 2014.

[18] I. Kontoyiannis and M. Skoularidou, “Estimating the directed information and testing for causality,” *IEEE Transactions on Information Theory*, vol. 62, no. 11, pp. 6053–6067, Nov. 2016.

[19] C. Diks and J. DeGoede, *A general nonparametric bootstrap test for Granger causality*. Institute of Physics Publishing, 2001, in *Global Analysis of Dynamical Systems*.
