# Supplementary material for: SozRank: A new approach for localizing the epileptic seizure onset zone
Source: PLoS Comput Biol. 2018 Jan 30;14(1):e1005953. doi: 10.1371/journal.pcbi.1005953 (PMC5806930; doi:10.1371/journal.pcbi.1005953)
Supplement: S2 Text — (PDF) [file pcbi.1005953.s002.pdf]

S2 Text - Supporting Information for "SozRank: A new approach for localizing the epileptic seizure onset zone"

Yonathan Murin[1], Jeremy Kim[1], Josef Parvizi[2], Andrea Goldsmith[1]

[1] Department of Electrical Engineering, Stanford University, Stanford, CA, USA
[2] Department of Neurology & Neurological Sciences, Stanford University, Stanford, CA, USA

This document details the full seizure-onset-zone (SOZ) inference results for the different inference approaches discussed in Tables 2–4 of the main manuscript. The numbers of the inferred electrodes together with the localization maps provided in Figures 2–4 of the main manuscript, facilitate calculating any statistics for comparing the different localization approaches.

1 Detailed Results for Table 2 of the Main Manuscript

Table 1 below details the inferred electrodes for the *GC only* and the *DI only* approaches (see Table 2 of the main manuscript). These results were obtained using PageRank for estimating the SOZ from the graph and using $p_0 = 10$ and $p_1 = 5$ in the post-processing phase.

Table 1: **Inferred electrodes for the *GC only* and the *DI only* approaches**. ϕ denote the empty set, namely, no electrodes were estimated.

Patient	GC only	DI only
I001_P034_D01	23, 27, 29	23,29
Study_004-2	17	8,13,19
Study_006	7, 10, 15, 16, 23, 25	ϕ
Study_010	11, 20, 28, 37, 44	12, 20, 28, 37, 44
Study_016	ϕ	22
Study_017	ϕ	5
Study_020	6, 9, 22	14, 16, 17
Study_021	23, 47, 48	ϕ
Study_022	ϕ	4, 9, 15, 16
Study_023	2, 9, 10, 11, 17, 18, 26	2, 9, 10, 11, 17, 18, 19, 26
Study_027	3,5	2, 9
Study_033	29, 37, 38, 44, 45, 46, 53, 61	ϕ
Study_037	51, 55, 56, 60, 64	9, 28, 55, 56, 59, 64
HUP64_phaseII	15, 16, 23, 24, 31, 32	20, 23, 24, 31, 32, 34, 46, 59
HUP65_phaseII	11, 12, 19, 20, 27	12, 19, 20, 27
HUP68_phaseII	4, 10, 11, 12, 19, 48	4, 5, 10, 11, 12, 14, 15, 20
HUP70_phaseII	3, 13, 20, 23, 29, 33	13,20,22,23,29
HUP78_phaseII	55, 57	33, 34, 41, 42, 49, 54, 57
HUP87_phaseII	20, 22, 28, 29, 35, 36, 37	28, 29, 31, 36, 37, 63

2 Detailed Results for Table 3 of the Main Manuscript

Table 2 below details the inferred electrodes for the *Net-flow* and *Top 5%* estimation methods (see Table 3 of the main manuscript). The Net-flow results were obtained using $p_0 = 10$ and $p_1 = 5$ in the post-processing phase.

Table 2: **Inferred electrodes for the *Net-flow* and *Top 5%* estimation methods**. ϕ denote the empty set, namely, no electrodes were estimated.

Patient	Net-flow	Top 5%
I001_P034_D01	23,29	23, 27, 29
Study_004-2	7, 8,13, 19, 20	13, 19, 20
Study_006	1, 9, 20, 25, 48	1, 8, 9, 25
Study_010	12, 19, 20, 28, 37, 44	12, 20, 37, 44
Study_016	22	3, 22, 23
Study_017	3	4
Study_020	6,14,16,17	6, 14, 17
Study_021	23,30, 47, 48	23, 24, 32, 38
Study_022	4, 9, 15, 16	9, 15, 16
Study_023	2, 9, 10, 11, 17, 18, 19, 26	2, 10, 11, 17, 18
Study_027	2, 5, 9	2, 9, 24
Study_033	12, 30	12, 20, 23, 30, 31
Study_037	9, 28, 55, 56, 59, 64	9, 28, 55, 56, 64
HUP64_phaseII	31, 34	23, 24, 31, 34, 59
HUP65_phaseII	12, 20, 21, 27, 28, 29, 37	12, 20, 28, 29, 37
HUP68_phaseII	4, 10, 11, 12, 14, 15, 19, 20	4, 11, 12, 14, 15
HUP70_phaseII	13, 14, 20, 22, 23, 29, 55	13, 20, 22, 23, 29
HUP78_phaseII	33, 34, 57	33, 34, 41, 49, 57
HUP87_phaseII	24, 28, 29, 31, 36, 37, 63	28, 29, 36, 37, 63

3 Detailed Results for Table 4 of the Main Manuscript

Table 3 below details the inferred electrodes for the *5 seconds* and *20 seconds* windows (see Table 4 of the main manuscript). These results were obtained using PageRank for estimating the SOZ from the graph and using $p_0 = 10$ and $p_1 = 5$ in the post-processing phase.

Table 3: **Inferred electrodes for the *5 seconds* and *20 seconds* windows**. ϕ denote the empty set, namely, no electrodes were estimated.

Patient	5 seconds	20 seconds
I001_P034_D01	23, 29, 33	21, 23, 27, 29, 33
Study_004-2	13, 19, 26, 27	1, 2, 17, 19, 20
Study_006	32	7, 10, 15, 16, 17, 23, 25
Study_010	20, 28, 29, 30	9, 12, 19, 20, 28, 37
Study_016	22	13, 22
Study_017	ϕ	5
Study_020	14, 22	9, 14
Study_021	36, 37, 38	9
Study_022	3, 9, 16, 24	1, 9, 16
Study_023	10, 11, 17, 18, 19, 61	2, 9, 10, 11, 17, 18, 19
Study_027	9	2, 3, 9, 11
Study_033	18, 64	36, 37, 61
Study_037	9, 51, 55, 56, 64	28, 35, 51, 55, 56, 64
HUP64_phaseII	22, 23, 30, 31, 32, 46, 60	23, 46, 54
HUP65_phaseII	28, 35	12, 18, 19, 20, 27, 37
HUP68_phaseII	11, 15, 16, 25	15, 25
HUP70_phaseII	22, 23, 52, 53, 63, 64	21, 22, 23, 29, 43, 48, 63, 64
HUP78_phaseII	61	49, 50, 57, 58, 59, 60
HUP87_phaseII	29, 31, 36	29, 31
